# Can artificial intelligence accelerate the diagnosis of inherited retinal diseases? Protocol for a data-only retrospective cohort study (Eye2Gene)

**DOI:** 10.1136/bmjopen-2022-071043

**Published:** 2023-03-20

**Authors:** Quang Nguyen, William Woof, Nathaniel Kabiri, Sagnik Sen, Malena Daich Varela, Thales Antonio Cabral De Guimaraes, Mital Shah, Dayyanah Sumodhee, Ismail Moghul, Saoud Al-Khuzaei, Yichen Liu, Catherine Hollyhead, Bhavna Tailor, Loy Lobo, Carl Veal, Stephen Archer, Jennifer Furman, Gavin Arno, Manuel Gomes, Kaoru Fujinami, Savita Madhusudhan, Omar A Mahroo, Andrew R Webster, Konstantinos Balaskas, Susan M Downes, Michel Michaelides, Nikolas Pontikos, Eye2Gene Patient Advisory Group, Catherine Hollyhead

**Affiliations:** 1 UCL Institute of Health Informatics, University College London, London, UK; 2 UCL Institute of Ophthalmology, University College London, London, UK; 3 Moorfields Eye Hospital NHS Foundation Trust, London, UK; 4 Oxford Eye Hospital, Oxford, UK; 5 UCL Cancer Institute, University College London, London, UK; 6 Eye2Gene Patient Advisory Group, London, UK; 7 UCL Translational Research Office, University College London, London, UK; 8 UCL Department for Applied Health Research, University College London, London, UK; 9 National Institute of Sensory Organs, National Hospital Organization Tokyo Medical Center, Kankakuki Center, Meguro-ku, Tokyo, Japan; 10 Royal Liverpool and Broadgreen University Hospitals NHS Trust, Liverpool, UK

**Keywords:** STATISTICS & RESEARCH METHODS, OPHTHALMOLOGY, GENETICS

## Abstract

**Introduction:**

Inherited retinal diseases (IRD) are a leading cause of visual impairment and blindness in the working age population. Mutations in over 300 genes have been found to be associated with IRDs and identifying the affected gene in patients by molecular genetic testing is the first step towards effective care and patient management. However, genetic diagnosis is currently slow, expensive and not widely accessible. The aim of the current project is to address the evidence gap in IRD diagnosis with an AI algorithm, Eye2Gene, to accelerate and democratise the IRD diagnosis service.

**Methods and analysis:**

The data-only retrospective cohort study involves a target sample size of 10 000 participants, which has been derived based on the number of participants with IRD at three leading UK eye hospitals: Moorfields Eye Hospital (MEH), Oxford University Hospital (OUH) and Liverpool University Hospital (LUH), as well as a Japanese hospital, the Tokyo Medical Centre (TMC). Eye2Gene aims to predict causative genes from retinal images of patients with a diagnosis of IRD. For this purpose, 36 most common causative IRD genes have been selected to develop a training dataset for the software to have enough examples for training and validation for detection of each gene. The Eye2Gene algorithm is composed of multiple deep convolutional neural networks, which will be trained on MEH IRD datasets, and externally validated on OUH, LUH and TMC.

**Ethics and dissemination:**

This research was approved by the IRB and the UK Health Research Authority (Research Ethics Committee reference 22/WA/0049) ‘Eye2Gene: accelerating the diagnosis of IRDs’ Integrated Research Application System (IRAS) project ID: 242050. All research adhered to the tenets of the Declaration of Helsinki. Findings will be reported in an open-access format.

**STRENGTHS AND LIMITATIONS OF THIS STUDY**

- One of the largest databases in the world of patients with inherited retinal disease who have undergone genetic screening and modern retinal imaging, analysed using novel artificial intelligence approaches.
- Robust evaluation and external validation at three different sites of an artificial intelligence algorithm on the task of automatically identifying up to 36 distinct genes from retinal images in patient suspected to have an inherited retinal disease.
- Artificial intelligence performance is very dependent on the gene distribution of the training dataset, which is very imbalanced in the case for inherited retinal diseases, hence the need for external validation.

## Introduction

The retina is the light-sensitive tissue at the back of our eyes, which transforms light into electrical signals to the brain and is responsible for vision. The inherited retinal diseases (IRDs) are a group of diseases resulting from variation in proteins involved in retinal function. They represent the most common cause of blindness in young people in the UK and a leading cause of severe visual impairment and/or blindness in the working age population.^1^ IRDs affect more than 2 million people globally and over 1 in 3000 people in the UK.^2 3^

The age of disease onset varies with different IRDs, and patients usually have a progressive deterioration of their peripheral or central vision over several decades.^4^ Hence, it is important to identify an IRD at an early stage, so that patients can undergo proper characterisation of the disease accurately. Treatments are emerging for some IRDs, but most are gene-specific, requiring identification of the precise causative genetic mutation.^5 6^

Mutations in over 300 genes are associated with IRDs.^7 8^ Identifying the causative gene is the first step towards diagnosis, prognosis and treatment. Currently, IRDs are usually detected first by community opticians and referred to ophthalmology departments for retinal imaging and diagnosis with a subsequent referral to specialist centres, such as Moorfields Eye Hospital (MEH), for further imaging and a genetic test.

**Figure 1:**
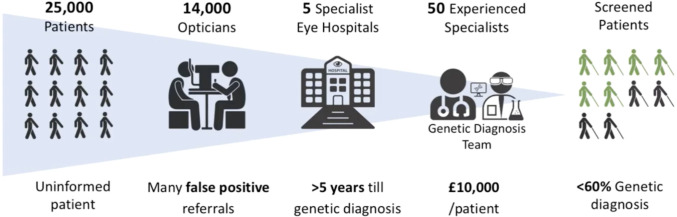
A summary of the inherited retinal disease patient population in the UK. On average, it takes over 5 years and approximately £10 000 for patients and families of patients to receive a final genetic diagnosis. Of the 30 000 individuals with inherited retinal disease, over one-third have not yet received a genetic diagnosis.

However, due to limitations in the availability of IRD clinical expertise, detection and diagnostic rates remain poor, with most individuals having to wait for an average of 5.6 years in the UK for a diagnosis.^9^ In addition, the diagnosis can cost the establishment £10 000 to obtain a final diagnosis for the patients and their families, starting from primary referral, to tertiary care, testing, investigation and genetic counselling.^1 10^ Hence, insufficient data on understanding of the disease prevalence and detection has contributed to insufficient funding available for testing of IRDs and associated counselling for patients and families. This delays development of possible treatment pathways and assistance with sight loss. As a result, a significant proportion of patients remain undiagnosed (figure 1).

The proposal here is to prepare images of historical IRD participant retinal scans (datasets) from eye hospitals located in the UK and in Japan:

- MEH.
- Oxford University Hospital (OUH).
- Liverpool University Hospital (LUH).
- Tokyo Medical Centre (TMC).

**Figure 2:**
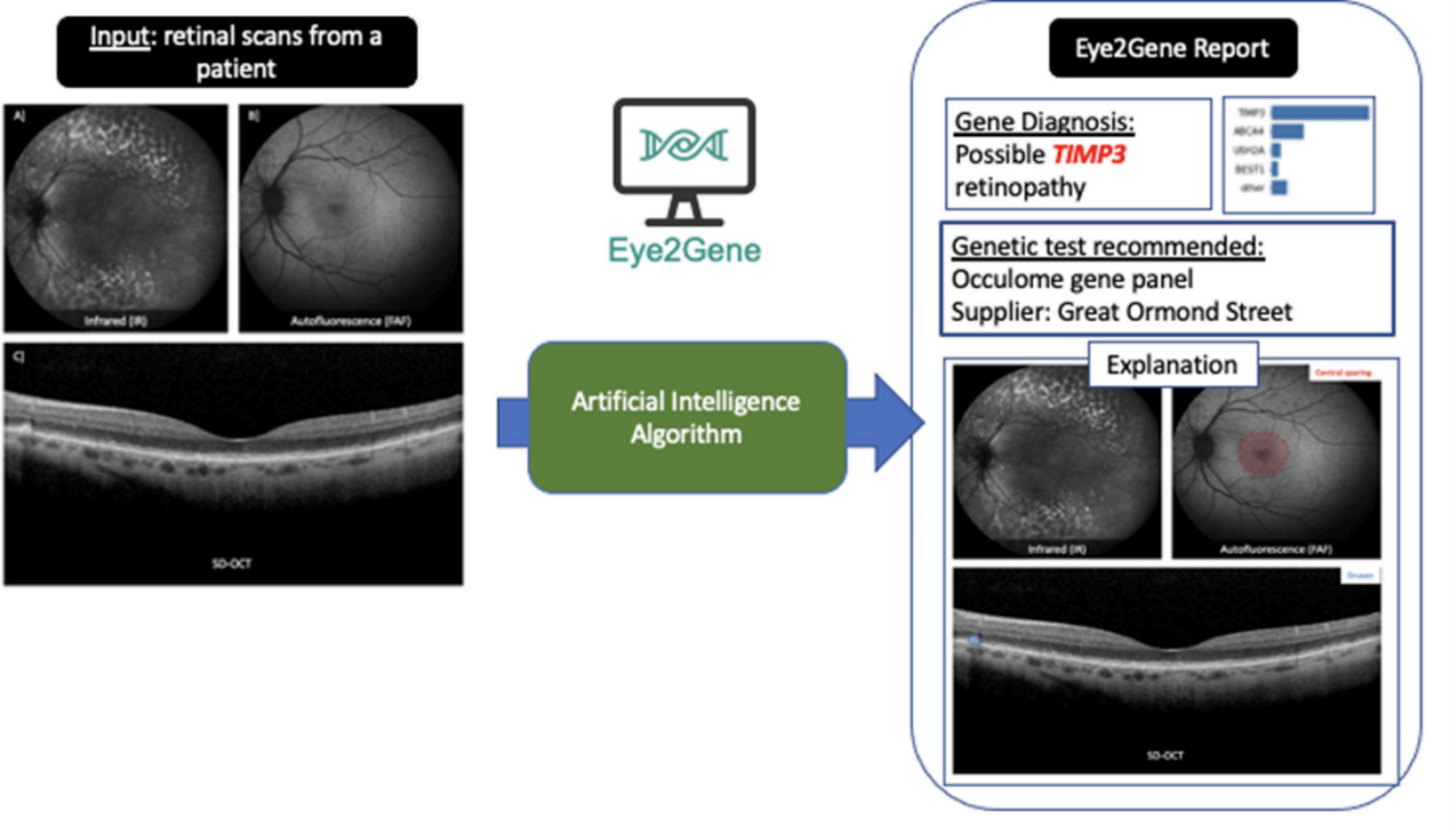
Eye2Gene supports the three main retinal imaging modalities: (A) Infrared (IR), (B) Autofluorescence (FAF) (C) Spectral domain optical coherence tomography (SD-OCT). FAF, fundus autofluorescence.

Retinal scan datasets will be used to benchmark, train and test Eye2Gene, a deep-learning algorithm designed to detect and diagnose IRDs from a participant’s retinal scan (figure 2).

### Aims and objectives

The aim of Eye2Gene is to provide detection and assist in diagnosis of IRDs through non-specialist centres within months instead of years. Eye2Gene does not aim to replace molecular diagnosis obtained through genetic testing, but it serves to narrow down the possibilities of genetic diagnosis based on imaging features, so that an early decision regarding patient care can be taken, and further testing offered after careful discussion with all stakeholders. It will also act as a tool for dissemination of expert IRD knowledge locally across the National Health Service (NHS).

By increasing the diagnostic rate for IRDs at a decreased cost, and by offering equitable access to a genetic diagnosis, the anticipated impacts for participants are:

- Improved health outcomes.
- Earlier clinical diagnosis.
- Personalised treatment plans (emerging treatments or clinical trials).
- Better understanding of the condition, its prognosis and its heritability for family planning.

For the NHS:

- Improved operational efficiency both for the prescription and interpretation of genetic tests.
- Increased genetic diagnostic rate at eye hospital.
- Reduced economic burden by not needing to test large gene panels or whole genome in every case.

The broad aim is to address the evidence gap in IRD diagnosis with an artificial intelligence (AI) algorithm, Eye2Gene, to accelerate and increase availability of a specialist IRD diagnostic service at point of care.

Our primary objectives are training and further validation of Eye2Gene on independent datasets from three external sites: OUH, LUH and TMC, which include:

- To refine and improve our model, particularly with respect to rarer genes.
- To provide explainability by identifying segmented IRD-specific features in classified images.
- To investigate and develop saliency maps for our networks.
- To validate Eye2Gene on external datasets to ensure it performs consistently well in different contexts (ie, that the model is generalisable).

Our secondary objectives are:

- To provide explainability by accurately identifying specific abnormalities (IRD-specific features) in retinal scans.
- To lay the groundwork for development of Eye2Gene into a medical device.

## Methods and analysis

### Work plan and timelines for delivery

**Figure 3:**
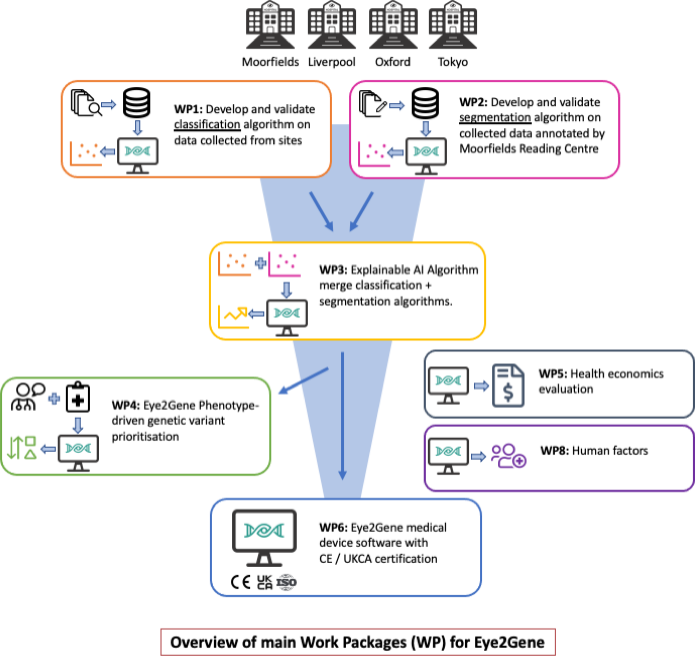
An overview of main WP for Eye2Gene. AI, artificial intelligence.

Eye2Gene project will be divided into eight work packages (WP) (illustrated in figure 3).

#### WP1: development of classification algorithm

This will involve developing a Convolutional Neural Network (CNN) model that can generalise to the N most common IRD genes at Moorfields and provide a top-five accuracy of at least 88%. In particular, we will focus on achieving high per-gene accuracy for the rarer genes (which the current iteration of the model currently underpredicts). In addition, part of this milestone will be to establish the value of N (the number of genes covered by the model), which we will pick based on all the data available across the four sites. We will assume N to be at least 10 for now, as this covers 70% of IRD cases and will be represented in the datasets of the four centres

#### WP2: development of segmentation algorithm

This will involve the manually curated and segmented dataset provided by the Moorfields Reading Centre IRD Segmentation Team. The team will consist of graders and software developers under the lead of Dr Balaskas at the Moorfields Reading Centre. These segmented IRD datasets will be useful for the training of multiple AI algorithms including Eye2Gene. These will include a total of 14 retinochoroidal features detectable by spectral domain optical coherence tomography (SD-OCT), infrared reflectance (IR) or short-wavelength fundus autofluorescence (FAF) or both, as well as their location, shape and distribution. A segmentation algorithm based on U-Net^11^ will be developed using this dataset.

#### WP3: development of explainable AI algorithm

By combining the output of the classification algorithm (WP1) with the segmentation/classification algorithms (WP2), we will build an explainable AI algorithm that combines accuracy (WP1) and explainability (WP2). The final output of these models will be combined in a multinomial logistic regression with additional optional inputs such as age, sex, ethnicity and mode of inheritance, to enhance predictive power. We will also be continuing to investigate and improve saliency maps for our models, and other explainability measures such as model confidence scores.

#### WP4: phenotype-driven genetic variant prioritisation

Deriving gene score based from the Eye2Gene classification gene probability from WP3. Also segmented IRD-features may be translated to Human Phenotype Ontology (HPO) terms in order to support HPO-base phenotype prioritisation using approaches such as Exomiser.^12^ We will assess the utility of Eye2Gene for phenotype-driven variant prioritisation to help solve cases with multiple candidate variants. This will fulfil the ACMG annotation guidelines PP4 criteria, namely that the patient’s phenotype or family history is highly specific for a disease with a single genetic aetiology.^13^

#### WP5: health economic evaluation

Health economic evaluation comparing the current treatment process to that of Eye2Gene will be conducted. The evaluation will consider two treatment pathways (standard care and the use of Eye2Gene), and will model resource use and cost, including the cost of validation, the cost of genetic tests, the time to find the genetics diagnosis (staff time) and the estimated cost of misdiagnosis, as well as the outcomes of standard and early diagnosis.

#### WP6: Eye2Gene medical software

Once we have completed the prototype as part of WP3, a software consultancy company (Phenopolis) will, under the oversight of regulatory consultants and the UCL Translational Research Office, develop Eye2Gene as medical device software following a QMS approach. In the first instance, the software will be developed to be hosted on a server that will likely be cloud based.

#### WP7: patient and public involvement

Patient advisory group (PAG) will feed into the decision making and the dissemination of results. The PAG will meet three times a year (January, May and September), each meeting will be 90 min and feed directly into the input of Eye2Gene. During this process, any risks raised by participants will be added to the risk register for the QMS.

#### WP8: human factors

User experience, usability and accessibility research will underpin the development of Eye2Gene. Following completion of WP3, we will have a working version of Eye2Gene to explore human factors around user expectations and experience.

### Study design and population

This is an investigation aiming to develop an AI software as a medical device. It is a data-only retrospective cohort study that will use images (retinal scans), associated scan-specific (such as laterality, scan date and modality) and participant-specific (such as molecular diagnosis, mode of inheritance, age and ethnicity) labels.

The study population includes data from participants that have received an IRD diagnosis, which has been molecularly confirmed via means of genetic test and have had retinal scans acquired using the Spectralis from Heidelberg Engineering (Dossenheim, Germany) with one of the following imaging modalities: IR, SD-OCT and FAF.

The study population at MEH has been derived by querying the OpenEyes Electronic Health Record (EHR) for IRD participants with a known genetic diagnosis and joining it up to the imaging databases of retinal scans (Heidelberg Medical Image Database) on hospital numbers. This enabled inclusion of all participants at MEH with an IRD who have both a genetic diagnosis and retinal scans available.

The study populations at OUH, LUH and TMC have been estimated based on information provided by the respective Principal Investigators, Prof Downes, Dr Madhusudhan and Prof Fujinami. This information has also been obtained by querying their local EHR databases and joining the dataset from the imaging database by hospital number.

### Derivation of sample size

The target sample size of 10 000 participants has been derived based on the number of participants with IRD at the three UK eye hospitals participating in this study (MEH, OUH and LUH), as well as a Japanese hospital, the TMC. Given the rare nature of IRDs and that the study works on retrospectively collected anonymised data, we are targeting the largest datasets available in the UK.

The 36 most common genes have been selected as these should have enough training examples to ensure at least 10 example images for each fold, when split into 5 folds (after removing an initial held-out participant set). This is to ensure a meaningful amount of test data for each class per-fold when performing a five-fold cross-validation study. This also ensures at least 40 training images per class for each split, which is about the minimum number of training examples with which a CNN can still achieve good results.^14^

### Data acquisition

**Figure 4:**
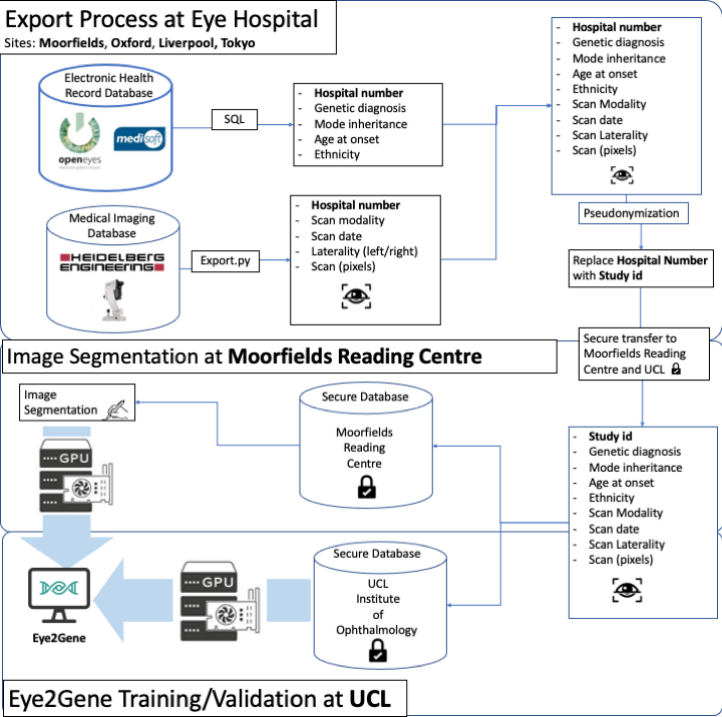
A data flow diagram summarising the extraction of data from Moorfields eye hospital and the external sites (OUH, LUH and TMC); secure transfer to the Moorfields eye hospital and UCL secure databases; and processing, to train and validate the Eye2Gene system. LUH, Liverpool University Hospital; OUH, Oxford University Hospital; TMC, Tokyo Medical Centre.

Participants will be identified by the care team of the respective site PIs by searching their medical records for patients who have received a molecularly confirmed genetic diagnosis for IRD. Data from MEH will be obtained by searching the EHR (OpenEyes) for participants with genetic reports entered in the EHR. The hospital numbers for these participants will be extracted and cross-referenced with the hospital numbers extracted from the imaging database, as shown in figure 4. A similar approach will be undertaken at LUH, OUH and TMC to link the imaging data to the genetic reports and other associated metadata (age, mode of inheritance and ethnicity) using the respective medical records in those sites and collating information from various spreadsheet, as needed.

Participants’ data extracted from medical records and imaging databases at each site will be used to produce a list of images labelled with genes and metadata, where available. Data will be pseudonymised by the respective clinical teams, assigning a unique study ID to each patient, and keeping the link between each study ID and original hospital number at each of the respective sites. The study team working on developing the AI algorithm at UCL will not have access to the original hospital IDs. Following export, the images will be quality controlled as described in the Inclusion criteria section below and uploaded, for each patient, to the Moorfields Reading Centre data-transfer portal secure database (grading.readingcentre.org).

Note that data collection for this study at each site is often an involved process since the data requires preparation, which needs to be overseen and carefully quality controlled by the site PI. First, patient genetic information is not always in the EHR in a research-ready format but instead located in study spreadsheets. Therefore, various spreadsheets containing participant-level information including demographics and clinical information such as genetic diagnosis and phenotype, may need to be collated. Once the participant information has been prepared, their corresponding scans need to be extracted from the Heyex medical imaging database. Since most sites lack a Vendor Neutral Archive (unfortunately these are still rare in ophthalmology), this process requires cross-referencing of scans to participant, extracting them from the Heyex database as E2E files one patient at a time, and uploading them to the Moorfields Reading Centre data-sharing platform (grading.readingcentre.org). These scans are then converted to an open format so they can be processed by the AI or annotated as part of WP2.

### Consent/consent exemptions

The project is limited to the use of previously collected, non-identifiable information. As only anonymised clinical data will be made available to the research team and no study procedures will be carried out as part of this study, informed consent will not be separately sought from participants. However, consent will be obtained from the participants of the human factors research conducted as part of WP8 to gather user feedback on Eye2Gene.

### Inclusion criteria

There will be no age restrictions for participants contributing data to train Eye2Gene, however, it is anticipated that most will be over the age of 18. The inclusion criteria require participants to have both a confirmed IRD genetic diagnosis available that conforms to criteria (A) below and retinal imaging scan data available that conforms to criteria (B) below.

#### Criteria for IRD genetic diagnosis

- An IRD genetic diagnosis consists of the identification of the IRD gene thought to be associated with the IRD condition of the participant.
- An IRD genetic diagnosis will often include the specific genetic variations which are thought to cause the disease.
- The IRD genetic diagnosis may have been conducted via a clinical NHS genetic testing service or through a research study.
- Both sources will be included in this study.

#### Criteria for retinal imaging scan data

Retina imaging scans will have been acquired with a medical imaging device (such as the Spectralis, Heidelberg) fixated on the macula and may belong to one of the following three categories:

- FAF.
- IR.
- SD-OCT.

Image quality will be an important factor to consider. In order to assess image quality objectively, image quality scores such as the Blind Referenceless Image Spatial Quality Evaluator^15^ image quality score will be applied. The criteria currently applied for image quality at MEH have been summarised in table 1. These scan quality thresholds will be reviewed and potentially adjusted depending on the data quality available across sites.

**Table 1:** Scan quality criteria for images obtained at MEH to maintain minimum standards of inclusion into the study

Quality of the FAF scans	Quality of the IR scans	Quality of the OCT scans
BRISQUE score <120	BRISQUE score <80	BRISQUE score <150
Median intensity >0.05	Median intensity >0.1	Max intensity <1 OR mean intensity <0.2
‘Noise level metric’ (ie, sum of square differences compared with blurred image via 5×5 box filter) <2200		

BRISQUE, Blind Referenceless Image Spatial Quality Evaluator; FAF, fundus autofluorescence; IR, infrared reflectance; MEH, Moorfields Eye Hospital; OCT, optical coherence tomography.

### Exclusion criteria

Participants that do not have a confirmed IRD genetic diagnosis or no retinal imaging data available. No other exclusion criteria apply.

### Time period of data collection and follow-up

The data collection will happen in the first 2 years of the study (January 2022–January 2024) to obtain retrospective observational data from all four sites. There will be no follow-up as all data are collected retrospectively for participants that have already received a genetic diagnosis. Following lead-in times, including ethics approvals, contractual procedures and data sharing agreements, data collection from UK sites (MEH, OUH and LUH) started in June 2022 and is likely to finish towards December 2023. Due to the additional challenges surrounding international data sharing and transfer arrangements, data collection at the Japanese site (TMC) was delayed to December 2022 and consequently, is likely to complete in January 2024. For the reasons explained above in ‘Data Acquisition’, the data collection, although retrospective, is a lengthy process which should finish by the end of 2023. In addition, as part of WP2, there is also an additional manual process undertaken of manually grading scans which will likely continue in the background for the entire duration of the project.

### Description of collected data

Along with the gene diagnosis and the retinal scans, the following information will be collected where available:

- Site: MEH, OUH, LUH or TMC
- Scan metadata:
  - Laterality.
  - Scan modality.
  - Date scan was acquired.
- Participant demographic data:
  - Age when scan was acquired.
  - Biological sex.
  - Ethnicity.
- Clinical information pertinent to disease:
  - Mode of inheritance.
  - Age of onset.

All data will be consistently coded across sites and pseudonymised. A unique study ID will be assigned to each participant and the link between the study IDs and original hospital number identifiers will be kept at each of the respective sites and not shared with the research team.

### Deep learning protocols

A CNN^16^ will be used to classify the images. It will be trained on retinal images from patients with IRD labelled with the causative gene. The aim will be to input a previously unseen retinal image and output a prediction of the causative gene (WP1) (see online supplemental figure 1).

10.1136/bmjopen-2022-071043.supp1 Supplementary data

Next, a subset of scans will be manually annotated, as part of WP2. This data will be used to train a U-Net,^11^ a commonly used neural network architecture for image segmentation tasks (online supplemental figure 2). These will include a total of 14 retinochoroidal features detectable by SD-OCT or FAF or both, as well as their location, shape, and distribution.

Specifically, on SD-OCT, we will segment eight features:

- Drusen.
- Subretinal fluid.
- Intraretinal fluid (cysts).
- Subretinal hyper-reflective material.
- Ellipsoid zone loss.
- Retinal pigment epithelium loss.
- Choroidal hypertransmission.
- Foveal hypoplasia.

On FAF/IR we will segment six features:

- Hypo/hyperautofluorescence patterns.
- Drusen
- Flecks.
- Peripapillary sparing.
- Vessel attenuation.
- Foveal hypoautofluorescence loss.

On the other hand, development of Deep Neural Network will also involve the manually curated and segmented dataset provided by the Moorfields Reading Centre IRD Segmentation Team. The team will consist of four graders and two software developers under the lead of an IRD expert at Moorfields and the director of the Moorfields Reading Centre.

### Statistical methods and performance evaluation

We aim to develop a model that can generalise to the N most common IRD genes at Moorfields and provide a top-five accuracy of at least 95%. In particular, we will focus on per-gene accuracy for the rarer genes (which the current iteration of the model currently underpredicts). Gene-specific or phenotype-specific segmentation features will be delineated in the Moorfields dataset and will be internally validated by the clinical team using the Dice similarity coefficient score:^17^

$$DSC=\frac{2|A\cap B|}{|A|+|B|}$$

where A and B are the regions defined by the two annotated features, to assess overlap with manual segmentation. Images with Dice score over 0.8 will be selected for training and validation.

The final output of classification and segmentation models will be combined in a multinomial logistic regression with additional optional inputs such as age, sex, ethnicity and mode of inheritance, to enhance predictive power (online supplemental figure 3). We will also be continuing to investigate saliency maps for our models, and other explainability measures such as model confidence scores.

The algorithm will be externally validated on the multisite data. This might require further calibration of parameter weights for age, sex, ethnicity and mode of inheritance, per site. We will use top-one and top-five classification accuracy and mean per-gene defined as area under the receiver operator curve score as the metrics for evaluation. We will also review the interpretation of the output (segmentation and saliency maps) qualitatively as part of Humans Factors (WP8). The data are stored at https://grading.readingcentre.org and can be made available for viewing at the discretion of the chief investigator NP. The code can be found at https://github.com/Eye2Gene.

### Measures to avoid bias

Site, age, gender, ethnicity and mode of inheritance are all potential sources of bias. These will be fitted as extra covariables into the classification algorithm using a multinomial regression or another equivalent statistical method to avoid confounding. Since only retrospective data will be used in a first instance, there will be no ascertainment bias as the data will represent routine IRD department activity at the respective hospitals.

A known source of bias in the data which cannot be corrected is the inherent imbalance in the data: more common diagnosis versus rarer diagnosis. The implications of this is that Eye2Gene will tend to overpredict the common classes and underpredict the rare classes.

### Patient and public involvement

Participants will be engaged for service mapping; knowledge gathering; acceptability testing and questionnaire design. A number of activities planned for the PAG and charity partners include:

- Interviews and focus groups.
- Survey and report designs.
- Data monitoring advice.
- Dissemination of research.

We will attend and present at participant days organised by charity partners, including Stargardt’s Connected and RetinaUK. During these conferences, Eye2Gene will be presented to participants who will be invited to share their views on the project. In the third year, we will liaise with the Moorfields patient and public involvement (PPI) team to organise an Eye2Gene participant day. Participants will be invited to discuss their diagnostic experience and view a presentation of Eye2Gene.

As the project advances, participants will be given the opportunity to participate in one-to-one interviews and focus groups, aiming at an in-depth investigation of participant experience and feedback on Eye2Gene (WP8). During these interviews, participant experience surrounding the genetic diagnosis process will be further explored:

- Aspects of health psychological support and health anxiety.
- Participant expectations about AI.
- Education and genetic counselling
- Areas for improvement.

Exploration of participant preferences (WP5) in year 2 (2023) will be formally quantified using a discrete choice experiment to investigate participants’ preference in their diagnostic journey will help inform future research and implementation.

The chief investigator, NP, and coinvestigators, supported by a health psychologist collaborator, DS, with experience in qualitative research and genetic counsellors will gather patient feedback at various events. These include focus groups held once a year (September), a participant day in the third year (June 2024) and any others organised by the Moorfields PPI teams.

During these events, participant experiences surrounding the genetic diagnosis process will be further explored. In particular, aspects of health psychological support, doctor–participant communication when discussing diagnosis and health anxiety, and participant education and genetic counselling. We will seek to understand how the current genetic diagnosis service experience can be improved with Eye2Gene. Preliminary results of PPI engagement can be found in online supplemental appendix A.

## Ethics and dissemination

This research was approved by the IRB and the UK Health Research Authority (Research Ethics Committee (REC) reference 22/WA/0049) ‘Eye2Gene: accelerating the diagnosis of inherited retinal diseases’ Integrated Research Application System (IRAS) (project ID: 242050). All research adhered to the tenets of the Declaration of Helsinki. Findings will be reported in an open-access format.

Our PPI group will be involved in dissemination of the results. In addition, our IRD participants that are signed up to our mailing lists will be informed of our progress.

## Supplementary Material

Reviewer comments

Author's manuscript
