# Supplementary material for: Can artificial intelligence accelerate the diagnosis of inherited retinal diseases? Protocol for a data-only retrospective cohort study (Eye2Gene)
Source: BMJ Open. 2023 Mar 20;13(3):e071043. doi: 10.1136/bmjopen-2022-071043 (PMC10030964; doi:10.1136/bmjopen-2022-071043)
Supplement: Supplementary data [file bmjopen-2022-071043supp001.pdf]

APPENDIX

A. PPI - Contributions to research design from patients

Patients have helped design and review the study protocol and have accepted the research. Acceptability and feasibility have been assessed as part of Phase 1 conducted through patient days with Moorfields (HDRUK-funded) and focus groups (NIHR PPIE enabling fund). In 2019, two focus groups and a patient day were conducted to explore IRD patient needs, as part of the HDRUK-funded MyEyeSite project.

When patients were interviewed to assess acceptability and feasibility of Eye2Gene to assist IRD diagnosis, our mixed-methods research (Gilbert et al., 2022) found that 82% wanted to be engaged in managing their own health data. Reasons given included:

- "To obtain genetic testing information for an affected child, or for fertility/genetics counselling family planning".
- "To participate in an international clinical trial".
- "Out of curiosity or personal interest in my condition".
- "To support a claim for personal independence payment".
- "To share data with another hospital (e.g., for diagnosing deafness or for cancer treatment)".

Further to this, an NIHR RDS Enabling Involvement Fund (awarded on the 12th of August 2020) allowed for the recruitment of 6 patients to review the Eye2Gene project, in addition to a further 4 who volunteered and waived compensation.

Two teleconference events with focus groups were organised in 2020, to provide feedback on the Eye2Gene proposal and the research programme. The first was held on the 21st of August and attended by 5 participants. The second, held on the 3rd of September, was attended by a further 5 participants. Both meetings were summarised in note form and moderated by a health psychologist collaborator. The major outcomes of the focus groups were:

- Patients suggested changes to the text to improve readability to a lay audience
- Patients clarified their needs and expectations of the project
- Patients suggested extending Eye2Gene to advise on potential treatments

Five patients volunteered to collaborate on the project, and have committed to specific roles as part of the Patient Advisory Group (PAG)

SUPPLEMENTARY FIGURES

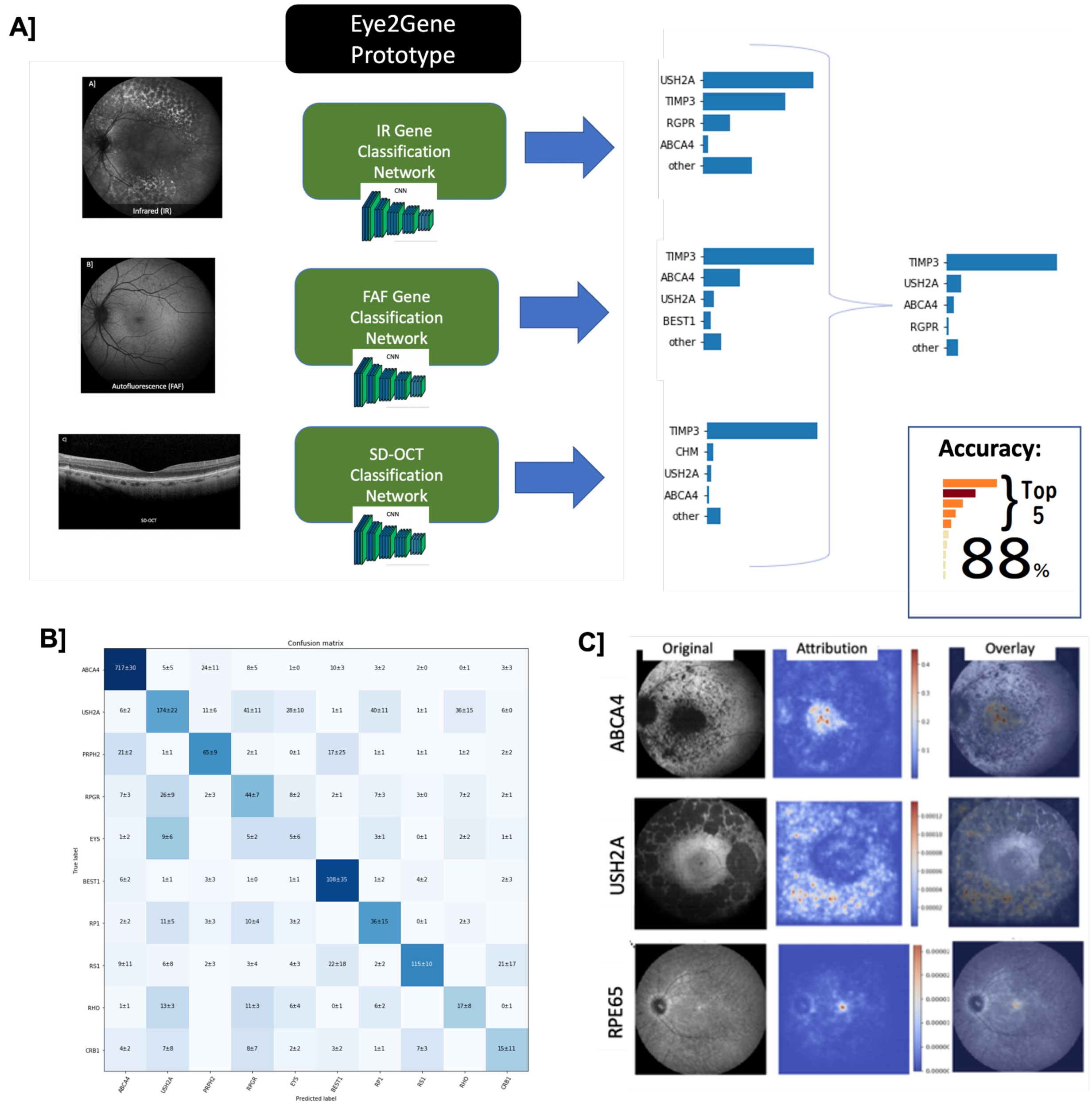

Supplementary Figure 1: A) The Eye2Gene prototype is able to provide an IRD-gene prediction given a retinal scan of one of the three imaging modalities (FAF; IR; and SD-OCT) (WP1). The top-5 accuracy of Eye2Gene is 88%. **B)** Confusion matrix indicating the misclassification errors for the top 10 genes. **C)** Attribution maps for FAFs indicate which pixels are deemed important by the network in reaching a classification. Cone-rod and macular dystrophies activate central pixels in the fovea such as ABCA4 and RPE65 whereas rod-cone dystrophies such as USH2A activate pixels in the periphery.

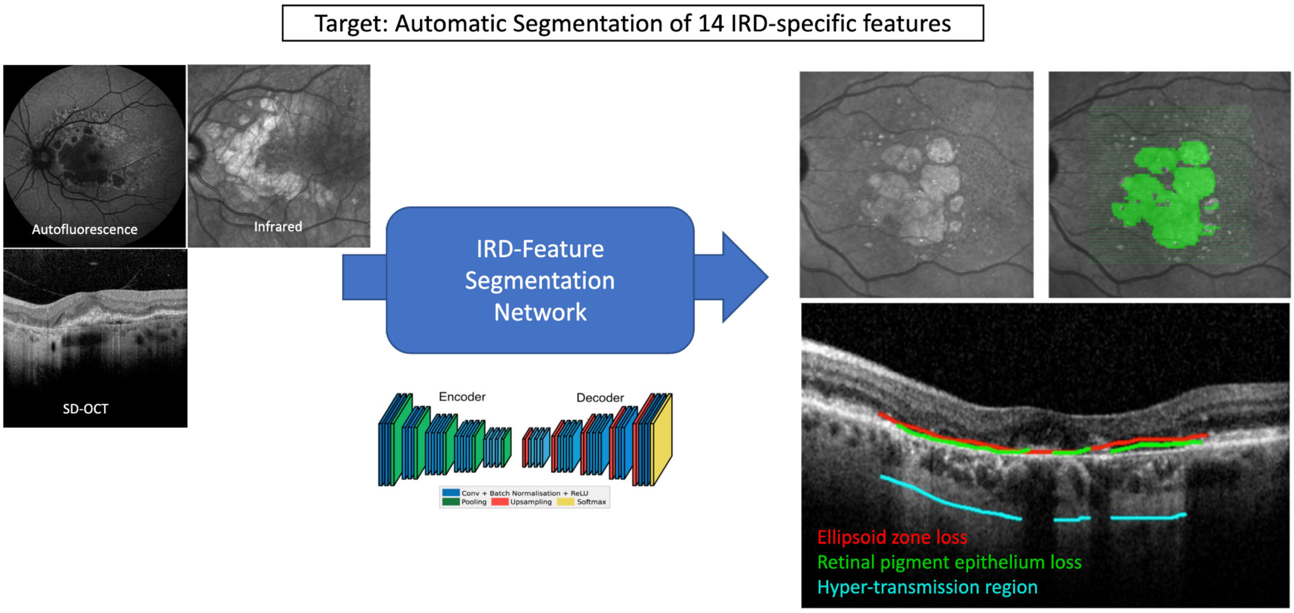

Supplementary Figure 2: The U-net architecture is characterized by an encoder-decoder structure. The encoder shares many similarities with classification networks, and aggregates information from a large spatial context into an abstract representation. From this abstract representation, the decoder subsequently reconstructs an image with the original resolution in which the output value for each pixel represents the segmentation label. This will be trained to segment the 14 features defined in WP2.

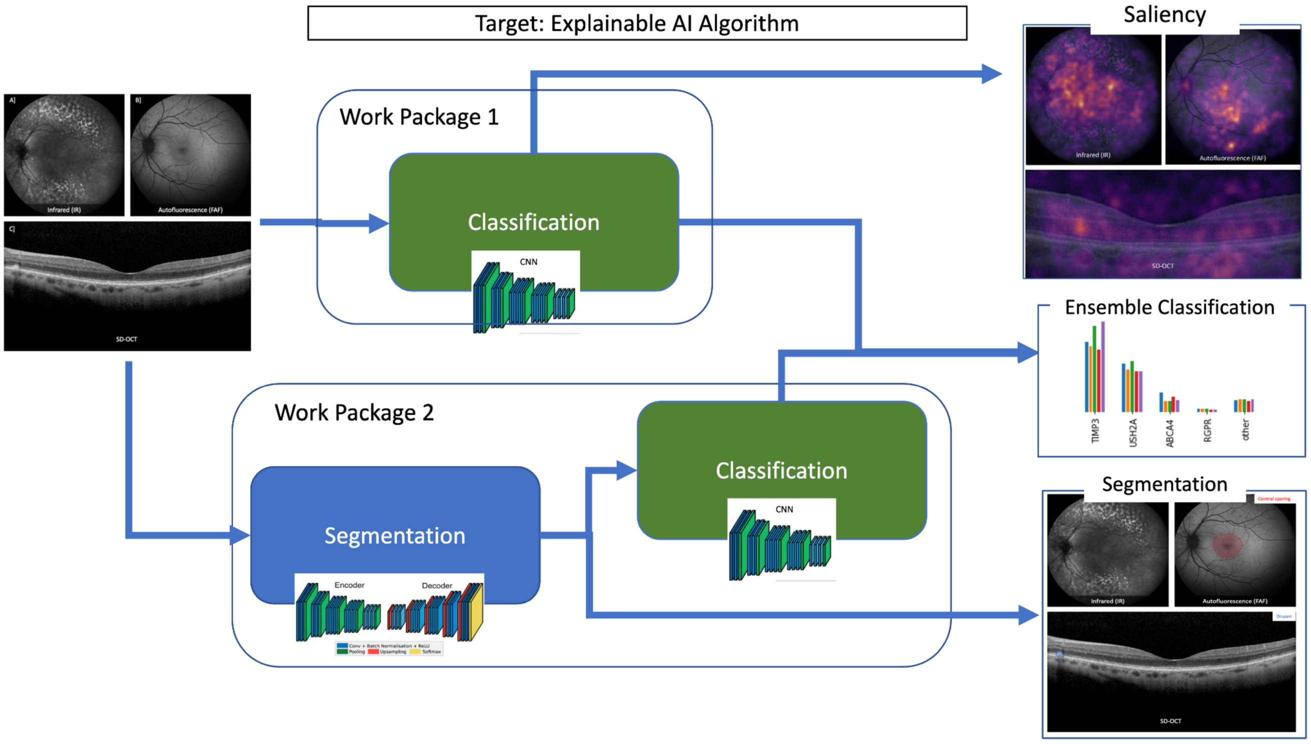

Supplementary Figure 3: By combining the classification network from WP1 with the segmentation network from WP2, Eye2Gene can provide highlight features used in the classification.
