# Supplementary material for: Can artificial intelligence accelerate the diagnosis of inherited retinal diseases? Protocol for a data-only retrospective cohort study (Eye2Gene)
Source: BMJ Open. 2023 Mar 20;13(3):e071043. doi: 10.1136/bmjopen-2022-071043 (PMC10030964; doi:10.1136/bmjopen-2022-071043)
Supplement: Author's manuscript [file bmjopen-2022-071043.draft_revisions.pdf]

BMJ Open is committed to open peer review. As part of this commitment we make the peer review history of every article we publish publicly available.

When an article is published we post the peer reviewers' comments and the authors' responses online. We also post the versions of the paper that were used during peer review. These are the versions that the peer review comments apply to.

The versions of the paper that follow are the versions that were submitted during the peer review process. They are not the versions of record or the final published versions. They should not be cited or distributed as the published version of this manuscript.

BMJ Open is an open access journal and the full, final, typeset and author-corrected version of record of the manuscript is available on our site with no access controls, subscription charges or pay-per-view fees (http://bmjopen.bmj.com).

If you have any questions on BMJ Open's open peer review process please email info.bmjopen@bmj.com

BMJ Open

Eye2Gene: accelerating the diagnosis of inherited retinal diseases with artificial intelligence - a study protocol

Journal:	*BMJ Open*
Manuscript ID	bmjopen-2022-071043
Article Type:	Protocol
Date Submitted by the Author:	17-Dec-2022

Complete List of Authors:	Nguyen, Quang; UCL Institute of Health Informatics,<br>Woof, William; Moorfields Eye Hospital NHS Foundation Trust; UCL Institute of Ophthalmology, Inherited Eye Diseases<br>Kabiri, Nathaniel; UCL Institute of Health Informatics<br>Sen, Sagnik; Moorfields Eye Hospital NHS Foundation Trust<br>Varela, Malena; Moorfields Eye Hospital NHS Foundation Trust; UCL Institute of Ophthalmology, Inherited Eye Diseases<br>Cabral De Guimaraes, Thales; UCL Institute of Ophthalmology, Inherited Eye Diseases; Moorfields Eye Hospital NHS Foundation Trust<br>Shah, Mital; Oxford Eye Hospital<br>Sumodhee, Dayyanah; Moorfields Eye Hospital NHS Foundation Trust<br>Moghul, Ismail; UCL Cancer Institute; Moorfields Eye Hospital NHS Foundation Trust<br>Al-Khuzaei, Saoud; Oxford Eye Hospital<br>Liu, Yichen; UCL Institute of Ophthalmology, Inherited Eye Diseases; Moorfields Eye Hospital NHS Foundation Trust<br>Hollyhead, Catherine; Eye2Gene Patient Advisory Group<br>Tailor, Bhavna; Eye2Gene Patient Advisory Group<br>Lobo, Loy; Eye2Gene Patient Advisory Group<br>Veal, Carl; Eye2Gene Patient Advisory Group<br>Archer, Stephen; Eye2Gene Patient Advisory Group<br>Furman, Jennifer; UCL<br>Arno, Gavin; UCL Institute of Ophthalmology, Inherited Eye Diseases<br>Gomes, Manuel; University College London, Department for Applied Health Research<br>Fujinami, Kaoru; Kankakuki Center<br>Madhusudhan, Savita; Royal Liverpool and Broadgreen University Hospitals NHS Trust<br>Mahroo, Omar; UCL Institute of Ophthalmology, Inherited Eye Diseases; Moorfields Eye Hospital NHS Foundation Trust<br>Webster, Andrew; University College London, Institute of Ophthalmology; Moorfields Eye Hospital NHS Foundation Trust<br>Balaskas, Konstantinos; Moorfields Eye Hospital NHS Foundation Trust, ; University of Manchester , School of Biological Sciences<br>Downes, Susan; Oxford Eye Hospital; Oxford Eye Hospital<br>Michaelides, Michael; University College London; University College London, Institute of Ophthalmology<br>Pontikos, Nikolas; University College London, Institute of Ophthalmology; Moorfields Eye Hospital NHS Foundation Trust

Keywords:	STATISTICS & RESEARCH METHODS, OPHTHALMOLOGY, GENETICS

SCHOLARONE™
Manuscripts

Eye2Gene: accelerating the diagnosis of inherited retinal diseases with artificial intelligence - a study protocol

Quang Nguyen[1,2,9], William Woof[1,2], Nathaniel Kabiri[1,2], Sagnik Sen[2], Malena Daich Varela[1,2], Thales Antonio Cabral de Guimarães[1,2], Mital Shah[3,4], Dayyanah Sumodhee[2], Ismail Moghul[2], Saoud Al-Khuzaei[3,4], Yichen Liu[1,2], Catherine Hollyhead[8], Bhavna Tailor[8], Loy Lobo[8], Carl Veal[8], Stephen Archer[8], Jennifer Furman[10], Gavin Arno[1,2], Manuel Gomes[16], Kaoru Fujinami[5,6], Savita Madhusadhan[7], Omar A Mahroo[1,2], Andrew R Webster[1,2], Konstantinos Balaskas[1,2], Susan Downes[3,4], Michel Michaelides[1,2], Nikolas Pontikos[1,2]*

*Corresponding author: n.pontikos@ucl.ac.uk

[1] University College London Institute of Ophthalmology, University College London, 11-43 Bath Street, London EC1V 9EL, UK
[2] Moorfields Eye Hospital, 162 City Road, London EC1V 2PD, UK
[3] Oxford Eye Hospital, John Radcliffe Hospital, Oxford OX3 9DU, UK
[4] Nuffield Department of Clinical Neurosciences, John Radcliffe Hospital, Oxford OX3 9DU, UK
[5] Laboratory of Visual Physiology, Division of Vision Research, National Institute of Sensory Organs, National Hospital Organization Tokyo Medical Center, Tokyo, Japan.
[6] Department of Ophthalmology, Keio University School of Medicine, Tokyo, Japan.
[7] St Paul's Eye Unit, The Royal Liverpool and Broadgreen University Hospitals, Liverpool L7 8XP, UK
[8] Eye2Gene Patient Advisory Group, London, UK
[9] University College London Institute of Health Informatics, 222 Euston Rd, London NW1 2DA, UK
[10] University College London Translational Research Office, 149 Tottenham Court Road, London W1T 7NF, UK
[11] University College London Institute of Epidemiology & Health, 1-19 Torrington Place, London WC1E 7HB, UK

ORCID:

Name	ORCID
Quang Nguyen	0000-0003-2665-3125
William Woof	0000-0001-7555-329X
Sagnik Sen	0000-0001-5835-5371
Malena Daich Varela	0000-0003-4960-4510
Thales Antonio Cabral de Guimarães	0000-0002-7936-6851
Mital Shah	0000-0001-7496-6121
Kaoru Fujinami	0000-0003-4248-0033
Savita Madhusudhan	0000-0002-8203-0929
Dayyanah Sumodhee	0000-0001-7274-6088
Ismail Moghul	0000-0003-3653-2327
Gavin Arno	0000-0002-6165-7888
Saoud Al-Khuzaei	0000-0002-2779-3202
Yichen Liu	0000-0002-9563-9151
Manuel Gomes	0000-0002-1428-1232
Omar A Mahroo	0000-0003-1254-0832
Andrew R Webster	0000-0001-6915-9560
Konstantinos Balaskas	0000-0003-2034-8920
Susan Downes	0000-0001-7373-2665
Michel Michaelides	0000-0002-1552-7046
Nikolas Pontikos	0000-0003-1782-4711

IRAS: 242050 Z6364106/2021/11/67

Word count: 3558

KEYWORDS: Artificial intelligence; Machine Learning; Retina; Eye Diseases, Hereditary; Blindness; Mass Screening; Vision Disorders

ABSTRACT

Introduction: Inherited retinal diseases (IRD) are a leading cause of visual impairment and blindness in the working age population. Mutations in over 300 genes have been seen to be associated with IRDs and identifying the affected gene in patients by molecular genetic testing is the first step towards effective care and patient management. However, genetics diagnosis is currently slow, expensive and not widely accessible. The aim of the current project is to address the evidence gap in IRD diagnosis with an AI algorithm, Eye2Gene, to accelerate and democratise the IRD diagnosis service.

Methods and Analysis: The study involves a target sample size of 10,000 participants, which has been derived based on the number of participants with IRD at the three leading UK eye hospitals: Moorfields Eye Hospital (MEH), Oxford University Hospital, and Liverpool University Hospital, as well as a Japanese hospital, the Tokyo Medical Centre (TMC). Eye2Gene aims to predict causative genes from retinal images of patients with a diagnosis of IRD. For this purpose, 36 most common causative IRD genes have been selected to develop a training dataset for the software to have enough examples for training and validation of each gene detection. The Eye2Gene algorithm is composed of multiple deep convolutional neural networks, which will be trained on MEH IRD datasets, externally validated on OUH, LUH and TMC.

Ethics and dissemination: This research was approved by the IRB and the UK Health Research Authority Research Ethics Committee.

ARTICLE SUMMARY

Strengths and limitations of this study

- One of the largest databases in the world of patients with IRD who have undergone genetic screening and modern retinal imaging, analysed using novel AI approaches.
- First application of AI to this task for 36 distinct genes with robust external validation at 3 different sites.
- AI performance is very dependent on the gene distribution of the training dataset which is very imbalanced for IRDs.

INTRODUCTION

Inherited Retinal Disease Situation

The retina is the light-sensitive tissue at the back of our eyes which transforms light into electrical signals to the brain and is responsible for vision. The inherited retinal diseases (IRDs) are a group of diseases resulting from variation in proteins involved in retinal function. They represent the most common cause of blindness in young people in the UK and a leading cause of severe visual impairment and/or blindness in the working age population [1]. IRDs affect more than 2 million people globally and over 1 in 3000 people in the UK [2,3].

The age of disease onset varies with different IRDs, and patients usually have a progressive deterioration of their peripheral or central vision over several decades [4]. Hence, it is important to identify an IRD at an early stage, so that patients can undergo proper characterisation of the disease accurately. Treatments are emerging for some IRDs, but most are gene-specific, requiring identification of the precise causative genetic mutation [5,6].

Mutations in over 300 genes are associated with IRDs [7,8]. Identifying the causative gene is the first step towards diagnosis, prognosis and treatment. Currently, IRDs are usually detected first by community opticians and referred to ophthalmology for retinal imaging and diagnosis with a subsequent referral to specialist eye hospitals, such as Moorfields Eye Hospital, for further imaging and a genetic test.

However, due to limitations in the availability of IRD clinical expertise, detection and diagnostic rates remain poor, with most individuals having to wait for an average of 5.6 years in the UK for a diagnosis [9]. In addition, the diagnosis can cost the establishment £10,000 to obtain a final diagnosis for the patients and their families, starting from primary referral, to tertiary care, testing, investigation and genetic counselling [1,10]. Hence insufficient data on understanding of the disease prevalence and detection has contributed to insufficient funding available for testing of IRDs and associated counselling for patients and families. This delays development of possible treatment pathways and assistance with sight loss. As a result, a significant proportion of patients remain undiagnosed (**Figure 1**).

The proposal herein is to prepare images of historical IRD participant retinal scans (datasets) from eye hospitals located in the UK and in Japan:

- Moorfields Eye Hospital (MEH)
- Oxford University Hospital (OUH)
- Liverpool University Hospital (LUH)
- Tokyo Medical Centre (TMC)

Retinal scan datasets will be used to benchmark, train and test Eye2Gene, a deep-learning algorithm designed to detect and diagnose IRDs from a participant's retinal scan (**Figure 2**).

Aims and Objectives

The aim of Eye2Gene is to provide detection and assist in diagnosis of IRDs through non-specialist centres within months instead of years. Eye2Gene does not aim to replace molecular diagnosis obtained through genetic testing, but it serves to narrow down the possibilities of genetic diagnosis based on imaging features, so that an early decision regarding patient care can be taken, and further testing offered after careful discussion with all stakeholders. It will also act as a tool for dissemination of expert IRD knowledge locally across the NHS.

By increasing the diagnostic rate for IRDs at a decreased cost, and by offering equitable access to a genetic diagnosis, the anticipated impacts for participants are:

- Improved health outcomes
- Earlier clinical diagnosis
- Personalised treatment plans (emerging treatments or clinical trials)
- Better understanding of the condition, its prognosis and its heritability for family planning

For the NHS:

- Improved operational efficiency both for the prescription and interpretation of genetic tests
- Increased genetic diagnostic rate at eye hospital
- Reduced economic burden by reducing the universality of genetic testing

The broad aim is to address the evidence gap in IRD diagnosis with an AI algorithm, Eye2Gene, to accelerate and increase availability of a specialist IRD diagnostic service at point of care.

Our primary objectives are training and further validation of Eye2Gene on independent datasets from three external sites: OUH, LUH and TMC, which include:

- To refine and improve our model, particularly with respect to rarer genes
- To provide explainability by identifying segmented IRD-specific features in classified images
- To investigate and develop saliency maps for our networks
- To validate Eye2Gene on external datasets to ensure it performs consistently well in different contexts (i.e that the model is generalisable)

Our secondary objectives are:

- To provide explainability by accurately identifying specific abnormalities (IRD-specific features) in retinal scans
- To lay the groundwork for development of Eye2Gene into a medical device

METHODS

Work plan and timelines for delivery

Eye2Gene project will be divided into eight Work Packages (WP) (illustrated in **Figure 3**).

WP1: Development of Classification Algorithm

This will involve developing a Convolutional Neural Network model that can generalise to the N most common IRD genes at Moorfields and provide a top-5 accuracy of at least 88%. In particular, we will focus on achieve high per-gene accuracy for the rarer genes (which the current iteration of the model currently underpredicts). Additionally, part of this milestone will be to establish the value of N (the number of genes covered by the model) which we will pick based on all the data available across the four sites. We will assume N to be at least 10 for now, as this covers 70% of IRD cases and will be represented in the datasets of the four centres

WP2: Development of Segmentation Algorithm

This will involve the manually curated and segmented dataset provided by the Moorfields Reading Centre IRD Segmentation Team. The team will consist of graders and software developers under the lead of Dr Balaskas at the Moorfields Reading Centre. These segmented IRD datasets will be useful for the training

of multiple AI algorithms including Eye2Gene. These will include a total of 14 retino-choroidal features detectable by SD-OCT or FAF or both, as well as their location, shape, and distribution. A segmentation algorithm based on U-Net [11] will be developed using this dataset.

WP3: Development of Explainable AI Algorithm

By combining the output of the classification algorithm (WP1) with the segmentation/classification algorithms (WP2), we will build an explainable AI algorithm that combines accuracy (WP1) and explainability (WP2). The final output of these models will be combined in a multinomial logistic regression with additional optional inputs such as age, sex, ethnicity, and mode of inheritance, to enhance predictive power. We will also be continuing to investigate and improve saliency maps for our models, and other explainability measures such as model confidence scores.

WP4: Phenotype-driven Genetic Variant Prioritisation

Deriving gene score based from the Eye2Gene classification gene probability from WP3. Also segmented IRD-features may be translated to Human Phenotype Ontology (HPO) terms in order to support HPO-base phenotype prioritisation using approaches like Exomiser. We will assess the utility of Eye2Gene for phenotype-driven variant prioritisation to help solve cases with multiple candidate variants. This will fulfil the ACMG annotation guidelines PP4 criteria (patient's phenotype or family history is highly specific for a disease with a single genetic aetiology)

WP5: Health Economic Evaluation

Health economic evaluation comparing the current treatment process to that of Eye2Gene will be conducted. The evaluation will consider two treatment pathways (standard care and the use of Eye2Gene), and will model resource use and cost, including the cost of validation, the cost of genetic tests, the time to find the genetics diagnosis (staff time), and the estimated cost of misdiagnosis, as well as the outcomes of standard and early diagnosis.

WP6: Eye2Gene Medical Software

Once we have completed the prototype as part of WP3, a software consultancy company (Phenopolis Ltd) will, under the oversight of the regulatory BCS Clinical Consulting and UCL Translational Research Office, develop Eye2Gene as medical device software following a QMS approach. In the first instance, the software will be developed to be hosted on a server that will likely be cloud-based.

WP7: Patient and Public Involvement

Patient Advisory Group (PAG) will feed into the decision making and the dissemination of results. The PAG will meet three times a year (January, May, September), each meeting will be 90 minutes and feed directly into the input of Eye2Gene. During this process, any risks raised by participants will be added to the risk register for the QMS.

WP8: Human Factors

User experience, usability and accessibility research will underpin the development of Eye2Gene. Following completion of WP3 we will have a working version of Eye2Gene to explore human factors around user expectations and experience.

Study Design and Population

This is an investigation aiming to develop an AI software as a medical device. It is a data-only retrospective cohort study that will utilise images (retinal scans), associated scan-specific (such as laterality, scan date and modality) and participant-specific (such as molecular diagnosis, mode of inheritance, age, and ethnicity) labels.

The study population includes data from participants that have received a IRD diagnosis, which has been molecularly confirmed via means of genetic test and have had retinal scans acquired with Optical Coherence Tomography (Spectralis Heidelberg Engineering, Dossenheim, Germany) and fundus autofluorescence camera.

The study population at MEH has been derived by querying the OpenEyes EHR for IRD participants with a known genetic diagnosis and joining it up to the imaging databases of retinal scans (Heidelberg Medical Image Database) on hospital numbers. This enabled inclusion of all participants at MEH with an IRD who have both a genetic diagnosis and retinal scans available.

The study populations at OUH, LUH and TMC have been estimated based on information provided by the respective PIs. This information has also been obtained by querying their local EHR databases and joining the dataset on the imaging database by hospital number.

Derivation of sample size

The target sample size of 10,000 participants has been derived based on the number of participants with IRD at the three leading UK eye hospitals participating in this study (MEH, OUH and LUH), as well as a Japanese hospital, the Tokyo Medical Centre (TMC). Given the rare nature of IRDs and that the study works on retrospectively collected anonymised data, we are targeting the largest datasets available in the UK.

The 36 most common genes have been selected as these should have enough training examples to ensure at least 10 example images for each fold, when split into 5 folds (after removing an initial held-out participant set). This is to ensure a meaningful amount of test data for each class per-fold when performing a 5-fold cross validation study. This also ensures at least 40 training images per class for each split, which is about the minimum number of training examples with which a CNN can still achieve good results [12].

Data Acquisition

Participants will be identified by the care team of the respective site PIs by searching their medical records for patients who have received a molecularly confirmed genetic diagnosis for IRD. Data from MEH will be obtained by searching the EHR (OpenEyes) for participants with genetic reports entered in the EHR. The hospital numbers for these participants will be extracted and cross-referenced with the hospital numbers extracted from the imaging database, as shown in **Figure 4**. A similar approach will be undertaken at LUH, OUH and TMC to link the imaging data to the genetic reports and other associated metadata (age; mode of inheritance; and ethnicity) using the respective medical records in those sites.

Participants' data extracted from medical records and imaging databases at each site will be used to produce a list of images labelled with genes and metadata, where available. Data will be pseudonymised by the respective clinical teams, assigning a unique study ID to each patient, and keeping the link between each study ID and original hospital number at each of the respective sites. The study team working on developing the AI algorithm at UCL will not have access to the original hospital IDs. Following export, the images will be quality controlled as described in the **Inclusion Criteria** section below.

Consent/Consent Exemptions

The project is limited to the use of previously collected, non-identifiable information. As only anonymised clinical data will be made available to the research team and no study procedures will be carried out as part of this study, informed consent will not be separately sought from participants. However, consent will be obtained from the participants of the human factors research conducted as part of WP8 to gather user feedback on Eye2Gene.

Inclusion Criteria

There will be no age restrictions for participants contributing data to train Eye2Gene, however it is anticipated that most will be over the age of 18. The inclusion criteria require participants to have both a confirmed IRD genetic diagnosis available that conforms to criteria (A) below and retinal imaging scan data available that conforms to criteria (B) below

(A) Criteria for IRD genetic diagnosis:

- An IRD genetic diagnosis consists of the identification of the IRD gene thought to be associated with the IRD condition of the participant.
- An IRD genetic diagnosis will often include the specific genetic variations which are thought to cause the disease.
- The IRD genetic diagnosis may have been conducted via a clinical NHS genetic testing service or through a research study.
- Both sources will be included in this study.

(B) Criteria for retinal imaging scan data:

Retina imaging scans will have been acquired with a medical imaging device (such as the Spectralis, Heidelberg) fixated on the macula and may belong to one of the following three categories:

- Fundus Autofluorescence
- Infrared
- Spectral Domain Optical Coherence Tomography

Image quality will be an important factor to consider. In order to assess image quality objectively, image quality scores such as the Blind Referenceless Image Spatial Quality Evaluator (BRISQUE) [13] image quality score will be applied. The criteria currently applied for image quality at MEH have been summarised in **Table 1.** These scan quality thresholds will be reviewed and potentially adjusted depending on the data quality available across sites.

Quality of the FAF scans	Quality of the IR scans	Quality of the OCT scans
- BRISQUE score < 120	- BRISQUE score < 80	- BRISQUE score < 150
- Median intensity > 0.05	- Median intensity > 0.1	- Max intensity < 1 OR mean intensity < 0.2
- "Noise level metric" (i.e. sum of square differences compared to blurred image via 5x5 box filter) < 2200		

Table 1: Scan quality criteria for images obtained at MEH to maintain minimum standards of inclusion into the study.

Exclusion Criteria

Participants that do not have a confirmed IRD genetic diagnosis or no retinal imaging data available. No other exclusion criteria apply.

Time period of data collection and follow-up

The data collection will happen in the first two years of the study to obtain retrospective observational data from all four sites. There will be no follow-up as all data is collected retrospectively for participants that have already received a genetic diagnosis.

Description of collected data

Along with the gene diagnosis and the retinal scans, the following information will be collected where available:

- Site: MEH, OUH, LUH or TMC
- Scan metadata:
  - Laterality
  - Scan modality
  - Date scan was acquired
- Participant demographic data:
  - Age when scan was acquired
  - Biological sex
  - Ethnicity
- Clinical information pertinent to disease:
  - Mode of inheritance
  - Age of onset

All data will be consistently coded across sites and pseudonymised. A unique study ID will be assigned to each participant and the link between the study IDs and original hospital number identifiers will be kept at each of the respective sites and not shared with the research team.

Deep Learning Protocols

A Convolutional Neural Network (CNN) [14] will be used to classify the images. It will be trained on retinal images from patients with IRD labelled with the causative gene. The aim will be to input a previously unseen retinal image and output a prediction of the causative gene (WP1) (see **Supplementary Figure 1**).

Next, a subset of scans will be manually annotated, as part of WP2. This data will be used to train a U-Net [11], a commonly used neural network architecture for image segmentation tasks (**Supplementary Figure 2**). These will include a total of 14 retino-choroidal features detectable by SD-OCT or FAF or both, as well as their location, shape, and distribution.

Specifically, on SD-OCT we will segment 8 features:

- Drusen
- Subretinal fluid
- Intraretinal fluid (cysts)
- Subretinal hyper-reflective material
- Ellipsoid zone loss
- Retinal pigment epithelium loss
- Choroidal hyper-transmission
- Foveal hypoplasia

On FAF/IR we will segment 6 features:

- Hypo/hyper autofluorescence patterns
- Drusen
- Flecks
- Peripapillary sparing
- Vessel attenuation
- Foveal hypo-autofluorescence loss

On the other hand, development of Deep Neural Network will also involve the manually curated and segmented dataset provided by the Moorfields Reading Centre IRD Segmentation Team. The team will consist of 4 graders and 2 software developers under the lead of an IRD expert at Moorfields and the director of the Moorfields Reading Centre.

Statistical methods and Performance Evaluation

We aim to develop a model that can generalise to the N most common IRD genes at Moorfields and provide a top-5 accuracy of at least 95%. In particular, we will focus on per-gene accuracy for the rarer genes (which the current iteration of the model currently underpredicts). Gene-specific or phenotype-specific segmentation features will be delineated in the Moorfields dataset and will be internally validated by the clinical team using the Dice similarity coefficient score [15]:

$$DSC = \frac{2\ |A \cap B\ |}{|A|\ +\ |B|}$$

where A and B are the regions defined by the two annotated features, to assess overlap with manual segmentation. Images with Dice score over 0.8 will be selected for training and validation.

The final output of classification and segmentation models will be combined in a multinomial logistic regression with additional optional inputs such as age, sex, ethnicity, and mode of inheritance, to enhance predictive power (**Supplementary Figure 3**). We will also be continuing to investigate saliency maps for our models, and other explainability measures such as model confidence scores.

The algorithm will be externally validated on the multisite data. This might require further calibration of parameter weights for age, sex, ethnicity, and mode of inheritance, per site. We will use top-1 and top-5 classification accuracy and mean per-gene defined as area under the receiver-operator curve (ROC AUC) score as the metrics for evaluation. We will also review the interpretation of the output (segmentation and saliency maps) qualitatively as part of Humans Factors (WP8).

Measures to avoid bias

Site, age, gender, ethnicity, and mode of inheritance are all potential sources of bias. These will be fitted as extra covariables into the classification algorithm using a multinomial regression or another equivalent statistical method to avoid confounding. Since only retrospective data will be used in a first instance there will be no ascertainment bias as the data will represent routine IRD department activity at the respective hospitals.

A known source of bias in the data which cannot be corrected is the inherent imbalance in the data: more common diagnosis vs rarer diagnosis. The implications of this is that Eye2Gene will tend to overpredict the common classes and underpredict the rare classes.

Patient And Public Involvement

Participants will be engaged for service mapping; knowledge gathering; acceptability testing; and questionnaire design. A number of activities planned for the PAG and charity partners include:

- Interviews and focus groups
- Survey and report designs
- Data monitoring advice
- Dissemination of research

We will attend and present at participant days organised by charity partners, including RetinaUK. During these conferences, Eye2Gene will be presented to participants who will be invited to share their views on the project. In the third year, we will liaise with the Moorfields PPI lead to organise an Eye2Gene participant day. Participants will be invited to discuss their diagnostic experience and view a presentation of Eye2Gene.

As the project advances, participants will be given the opportunity to participate in one-to-one interviews and focus groups, aiming at an in-depth investigation of participant experience and feedback on Eye2Gene (WP8). During these interviews, participant experience surrounding the genetic diagnosis process will be further explored:

- Aspects of health psychological support and health anxiety
- Participant expectations about AI
- Education and genetic counselling
- Areas for improvement

Exploration of participant preferences (WP5) in year two (2023) will be formally quantified using a Discrete Choice Experiment (DCE) to investigate participants' preference in their diagnostic journey will help inform future research and implementation.

The CI and co-investigators, supported by a health psychologist collaborator with experience in qualitative research and genetic counsellors will gather patient feedback at various events. These include focus groups held once a year (September), a participant day in the third year (June 2024), and any others organised by the INSIGHT hub or Moorfields PPI teams.

During these events, participant experiences surrounding the genetic diagnosis process will be further explored. In particular, aspects of health psychological support, doctor-participant communication when discussing diagnosis and health anxiety, and participant education and genetic counselling. We will seek to understand how the current genetic diagnosis service experience can be improved with Eye2Gene. Preliminary results of PPI engagement can be found in **Appendix A.**

STATEMENTS

Acknowledgments

As NIHR funded this study, publications will be published according to their guidelines. Moorfields Eye Charity has seed-funded part of this work, hence will be acknowledged in publications. Furthermore, the Archer family has also made a donation to the Eye2Gene project previously, hence will also be acknowledged.

Ethics and dissemination:

The Research Ethics Committee (REC) reference is 22/WA/0049 "Eye2Gene: accelerating the diagnosis of inherited retinal diseases" Integrated Research Application System (IRAS) (project ID: 242050). All research adhered to the tenets of the Declaration of Helsinki. All findings will be published in an open-access journal.

Data Statement

The data is stored at https://grading.readingcentre.org and can be made available for viewing at the request of the editors/reviewers. The code can be found at https://github.com/Eye2Gene.

Dissemination to the public

Our PPI group will be involved in dissemination of the results. Additionally, our IRD participants that are signed up to our mailing lists will be informed of our progress.

Competing interests statement

Neither the chief investigator (Dr Pontikos) nor any of the co-investigators have any direct personal involvement (financial, shareholder or personal) with the NIHR that may give rise to a conflict of interest.

As has been disclosed to UCL and NIHR, Dr Pontikos and Dr Moghul are shareholders at Phenopolis Ltd, which is one of the two subcontractors mentioned above. Phenopolis Ltd will provide professional software development services for Eye2Gene.

Funding statement

The work was funded by grant number AI_AWARD02488 from the National Institute for Health Research (NIHR) Biomedical Research Centre (BRC) at Moorfields Eye Hospital NHS Foundation Trust, and UCL Institute of Ophthalmology.

Author Contributions

The chief investigator NP conceptualised the Eye2Gene study, designed and developed the protocol. NP, QN and WW contributing to deep learning protocols. SS, TAC, MV, OM, MM, AW, KB, SD, SM, contributed to reviewing the clinical background. GA contributed to writing the phenotype-driven interpretation work package. MG contributed to writing the health economics work package. IM and MS contributed to writing the software as a medical device work package. DS, NK, WW and the patient advisory group contributed to writing the Patient and Public Involvement work package. DS contributed to writing the human factors work package. JF and SA contributed to writing the project management work package. NP contributed to all work packages. All authors contributed to critically reviewing and formatting the protocol. All authors approved the final version of the manuscript for submission for publication.

REFERENCES

[1] Galvin O, Chi G, Brady L, Hippert C, Del Valle Rubido M, Daly A, et al. The Impact of Inherited Retinal Diseases in the Republic of Ireland (ROI) and the United Kingdom (UK) from a Cost-of-Illness Perspective. Clin Ophthalmol 2020;14:707–19. https://doi.org/10.2147/OPTH.S241928.

[2] Berger W, Kloeckener-Gruissem B, Neidhardt J. The molecular basis of human retinal and vitreoretinal diseases. Prog Retin Eye Res 2010;29:335–75. https://doi.org/10.1016/j.preteyeres.2010.03.004.

[3] Hanany M, Rivolta C, Sharon D. Worldwide carrier frequency and genetic prevalence of autosomal recessive inherited retinal diseases. Proc Natl Acad Sci U S A 2020;117:2710–6. https://doi.org/10.1073/pnas.1913179117.

[4] Chen T-C, Huang D-S, Lin C-W, Yang C-H, Yang C-M, Wang VY, et al. Genetic characteristics and epidemiology of inherited retinal degeneration in Taiwan. NPJ Genom Med 2021;6:16. https://doi.org/10.1038/s41525-021-00180-1.

[5] Georgiou M, Fujinami K, Michaelides M. Inherited retinal diseases: Therapeutics, clinical trials and end points-A review. Clin Experiment Ophthalmol 2021;49:270–88. https://doi.org/10.1111/ceo.13917.

[6] Varela MD, de Guimaraes TAC, Georgiou M, Michaelides M. Leber congenital amaurosis/early-onset severe retinal dystrophy: current management and clinical trials. Br J Ophthalmol 2022;106:445–51. https://doi.org/10.1136/bjophthalmol-2020-318483.

[7] Duncan JL, Pierce EA, Laster AM, Daiger SP, Birch DG, Ash JD, et al. Inherited Retinal Degenerations: Current Landscape and Knowledge Gaps. Transl Vis Sci Technol 2018;7:6. https://doi.org/10.1167/tvst.7.4.6.

[8] Lázaro-Guevara JM, Flores-Robles BJ, Garrido K, Pinillos-Aransay V, Elena-Ibáñez A, Merino-Meléndez L, et al. Gene's hubs in retinal diseases: A retinal disease network. Heliyon 2018;4:e00867. https://doi.org/10.1016/j.heliyon.2018.e00867.
[9] The Lancet Diabetes Endocrinology. Spotlight on rare diseases. Lancet Diabetes Endocrinol 2019;7:75. https://doi.org/10.1016/S2213-8587(19)30006-3.
[10] Mendelian. A preliminary assessment of the potential impact of rare diseases on the NHS. 2018.
[11] Ronneberger O, Fischer P, Brox T. U-net: Convolutional networks for biomedical image segmentation. Lecture Notes in Computer Science (including subseries Lecture Notes in Artificial Intelligence and Lecture Notes in Bioinformatics), vol. 9351, Springer Verlag; 2015, p. 234–41. https://doi.org/10.1007/978-3-319-24574-4_28.
[12] Cho J, Lee K, Shin E, Choy G, Do S. How much data is needed to train a medical image deep learning system to achieve necessary high accuracy? ArXiv [CsLG] 2015.
[13] Mittal A, Moorthy AK, Bovik AC. Blind/Referenceless Image Spatial Quality Evaluator. 2011 Conference Record of the Forty Fifth Asilomar Conference on Signals, Systems and Computers (ASILOMAR), 2011, p. 723–7. https://doi.org/10.1109/ACSSC.2011.6190099.
[14] Lecun Y, Bengio Y, Hinton G. Deep learning. Nature 2015;521:436–44. https://doi.org/10.1038/nature14539.
[15] Dice LR. Measures of the Amount of Ecologic Association Between Species. Ecology 1945;26:297–302. https://doi.org/10.2307/1932409.

FIGURE LEGENDS

Figure 1: A summary of the inherited retinal disease patient population in the United Kingdom. On average, it takes over 5 years and approximately £10,000 for patients and families of patients to receive a final genetic diagnosis. Of the 30,000 individuals with inherited retinal disease, over a third have not yet received a genetic diagnosis.

Figure 2: Eye2Gene is an AI algorithm that rapidly recognises the gene associated with an inherited retinal disease, accelerating the genetic diagnosis. Eye2Gene supports the three main retinal imaging modalities: **(A)** Infrared (IR) **(B)** Autofluorescence (FAF) **(C)** Spectral domain optical coherence tomography (SD-OCT).

Figure 3: An overview of main work packages (WP) for Eye2Gene.

Figure 4: A data flow diagram summarising the extraction of data from Moorfields Eye Hospital and the external sites (OUH; LUH; and TMC); secure transfer to the Moorfields Eye Hospital and UCL secure databases; and processing, to train and validate the Eye2Gene system.

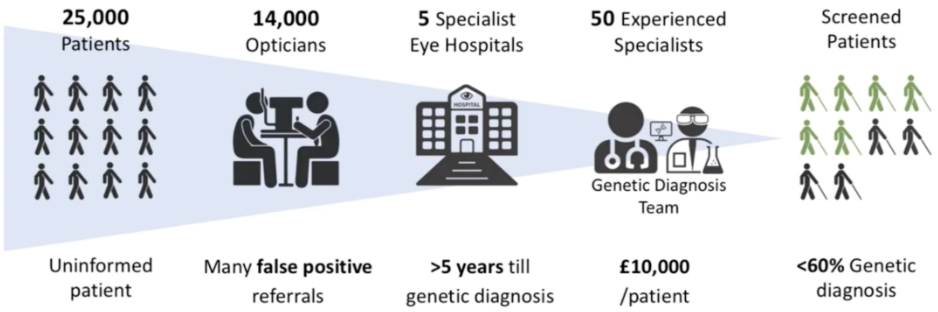

A summary of the inherited retinal disease patient population in the United Kingdom. On average, it takes over 5 years and approximately £10,000 for patients and families of patients to receive a final genetic diagnosis. Of the 30,000 individuals with inherited retinal disease, over a third have not yet received a genetic diagnosis.

383x136mm (144 x 144 DPI)

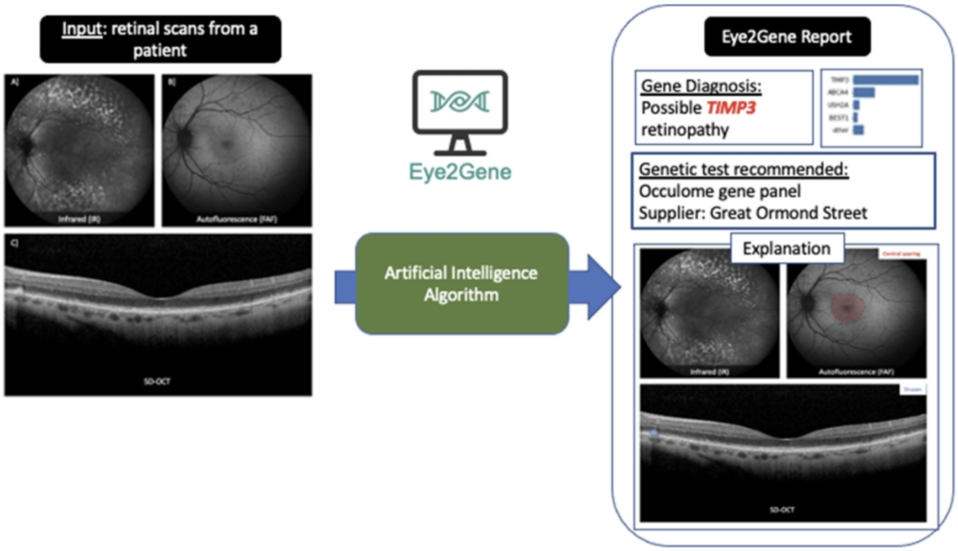

Eye2Gene is an AI algorithm that rapidly recognises the gene associated with an inherited retinal disease, accelerating the genetic diagnosis. Eye2Gene supports the three main retinal imaging modalities: (A) Infrared (IR) (B) Autofluorescence (FAF) (C) Spectral domain optical coherence tomography (SD-OCT).

524x301mm (144 x 144 DPI)

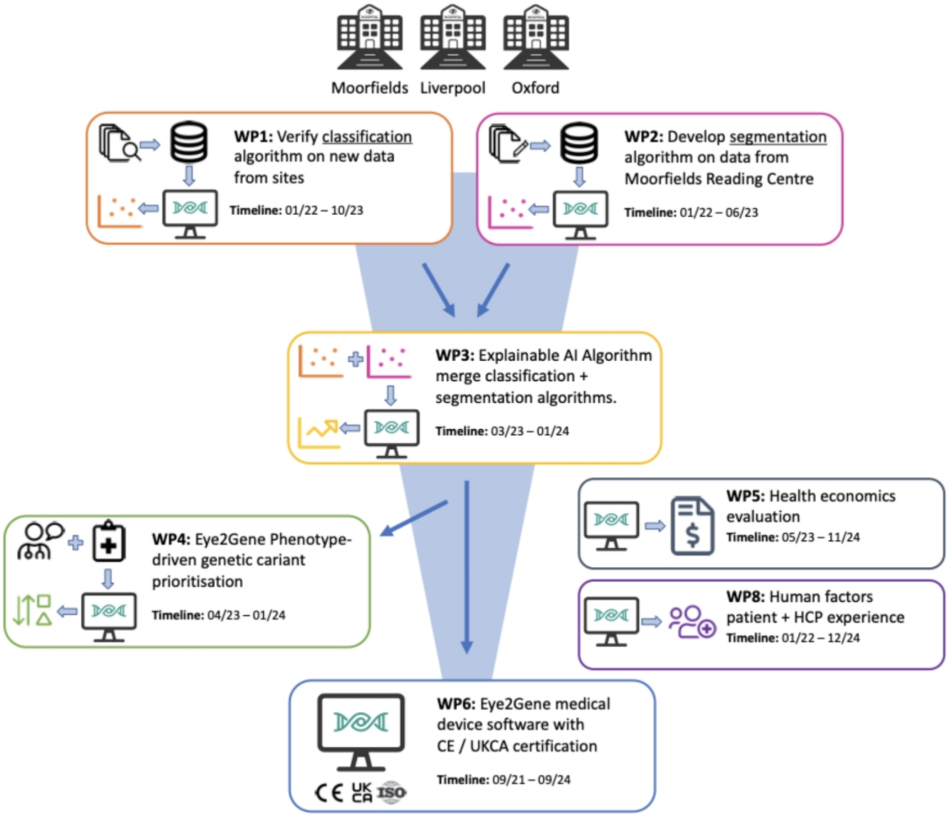

An overview of main work packages (WP) for Eye2Gene.

370x313mm (144 x 144 DPI)

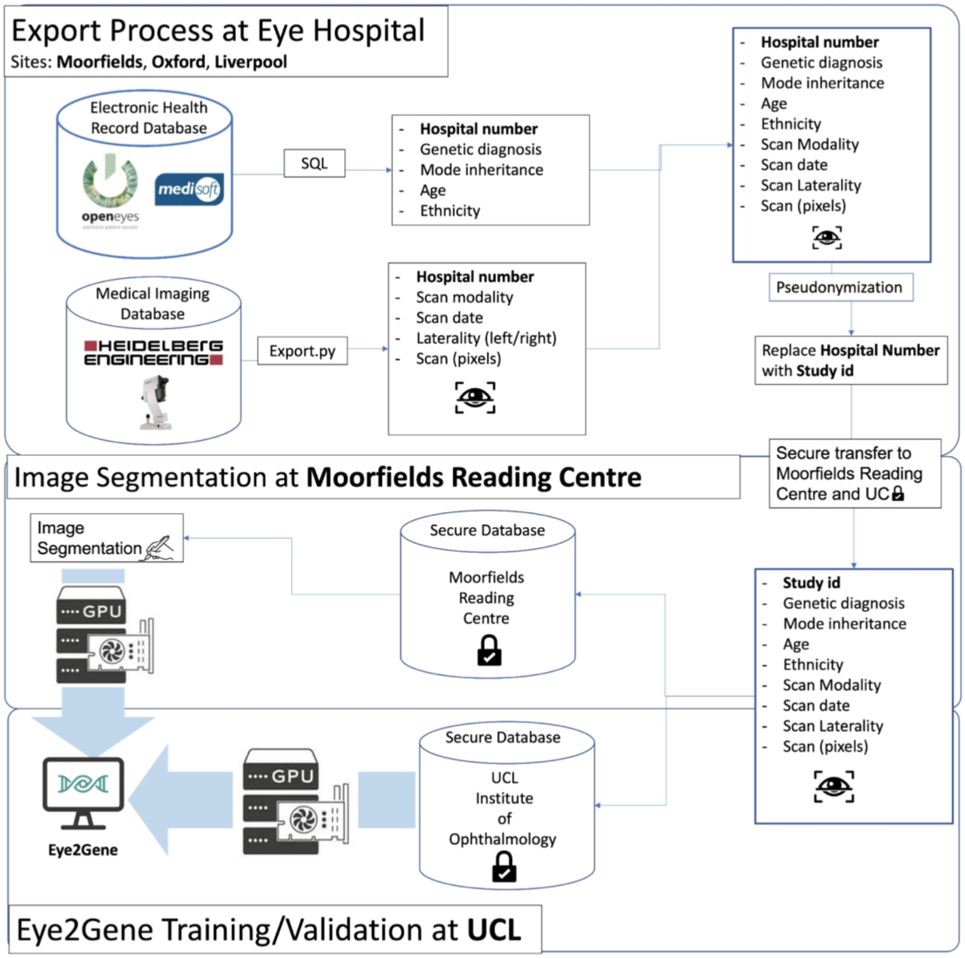

A data flow diagram summarising the extraction of data from Moorfields Eye Hospital and the external sites (OUH; LUH; and TMC); secure transfer to the Moorfields Eye Hospital and UCL secure databases; and processing, to train and validate the Eye2Gene system.

321x318mm (144 x 144 DPI)

APPENDIX

A. PPI - Contributions to research design from patients

Patients have helped design and review the study protocol and have accepted the research. Acceptability and feasibility have been assessed as part of Phase 1 conducted through patient days with Moorfields (HDRUK-funded) and focus groups (NIHR PPIE enabling fund). In 2019, two focus groups and a patient day were conducted to explore IRD patient needs, as part of the HDRUK-funded MyEyeSite project.

When patients were interviewed to assess acceptability and feasibility of Eye2Gene to assist IRD diagnosis, our mixed-methods research (Gilbert et al., 2022) found that 82% wanted to be engaged in managing their own health data. Reasons given included:

- “To obtain genetic testing information for an affected child, or for fertility/genetics counselling family planning”.
- “To participate in an international clinical trial”.
- “Out of curiosity or personal interest in my condition”.
- “To support a claim for personal independence payment”.
- “To share data with another hospital (e.g., for diagnosing deafness or for cancer treatment)”.

Further to this, an NIHR RDS Enabling Involvement Fund (awarded on the 12th of August 2020) allowed for the recruitment of 6 patients to review the Eye2Gene project, in addition to a further 4 who volunteered and waived compensation.

Two teleconference events with focus groups were organised in 2020, to provide feedback on the Eye2Gene proposal and the research programme. The first was held on the 21st of August and attended by 5 participants. The second, held on the 3rd of September, was attended by a further 5 participants. Both meetings were summarised in note form and moderated by a health psychologist collaborator. The major outcomes of the focus groups were:

- Patients suggested changes to the text to improve readability to a lay audience
- Patients clarified their needs and expectations of the project
- Patients suggested extending Eye2Gene to advise on potential treatments

Five patients volunteered to collaborate on the project, and have committed to specific roles as part of the Patient Advisory Group (PAG)

For peer review only

SUPPLEMENTARY FIGURES

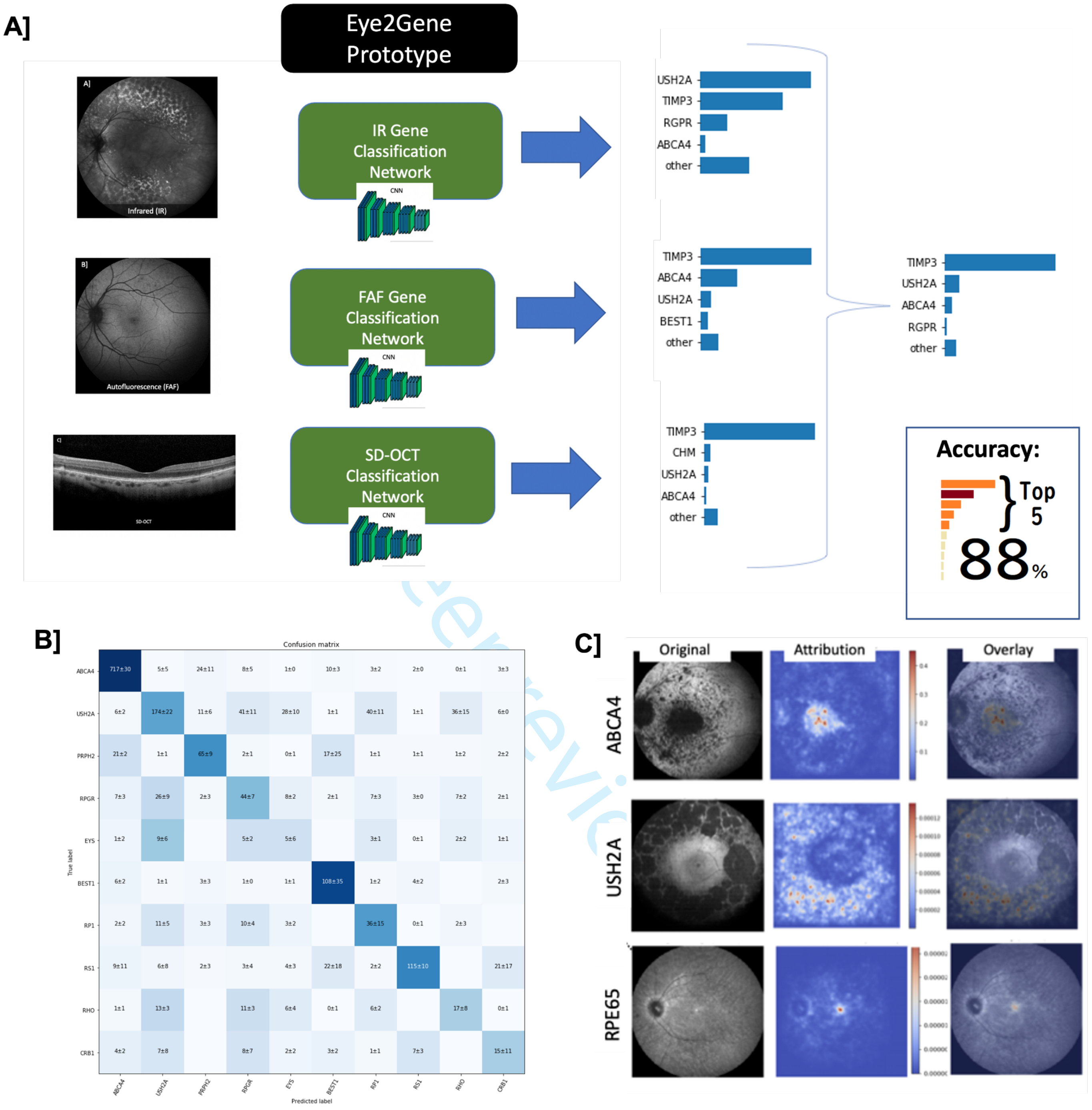

Supplementary Figure 1: A) The Eye2Gene prototype is able to provide an IRD-gene prediction given a retinal scan of one of the three imaging modalities (FAF; IR; and SD-OCT) (WP1). The top-5 accuracy of Eye2Gene is 88%. **B)** Confusion matrix indicating the misclassification errors for the top 10 genes. **C)** Attribution maps for FAFs indicate which pixels are deemed important by the network in reaching a classification. Cone-rod and macular dystrophies activate central pixels in the fovea such as ABCA4 and RPE65 whereas rod-cone dystrophies such as USH2A activate pixels in the periphery.

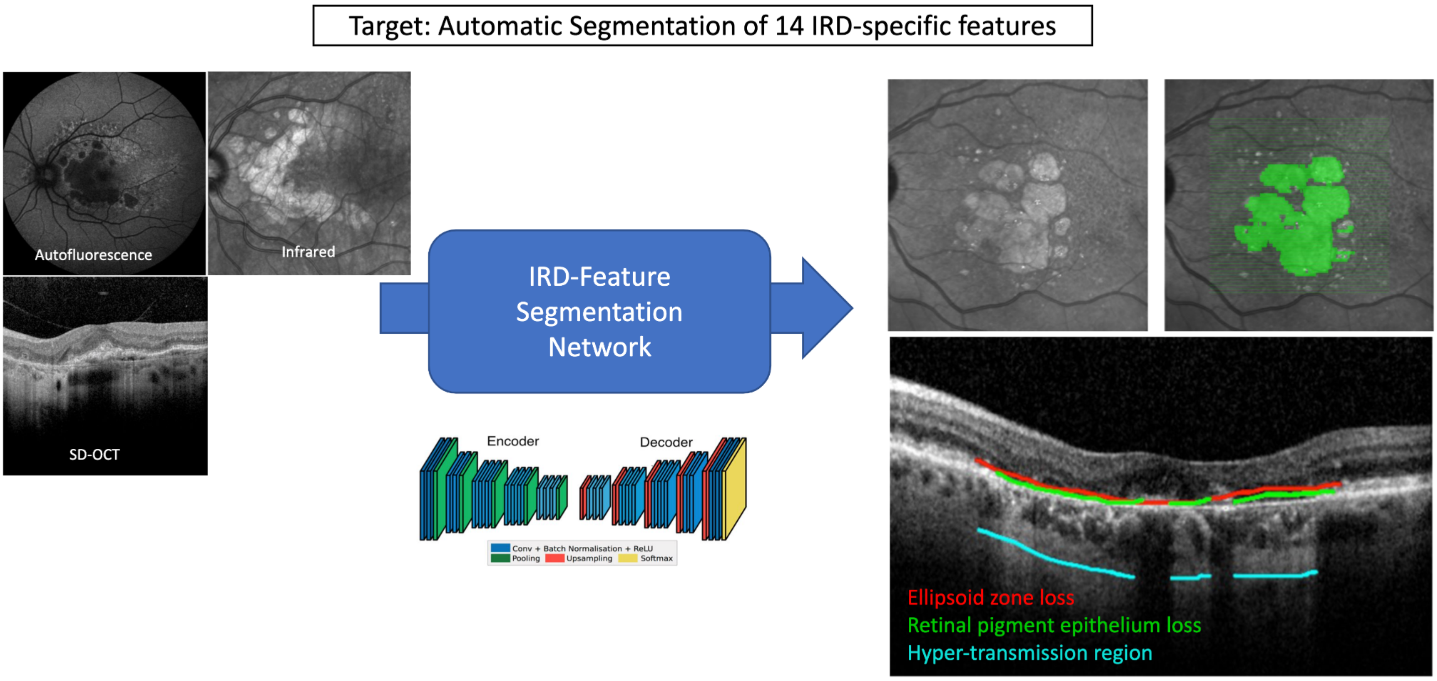

Supplementary Figure 2: The U-net architecture is characterized by an encoder-decoder structure. The encoder shares many similarities with classification networks, and aggregates information from a large spatial context into an abstract representation. From this abstract representation, the decoder subsequently reconstructs an image with the original resolution in which the output value for each pixel represents the segmentation label. This will be trained to segment the 14 features defined in WP2.

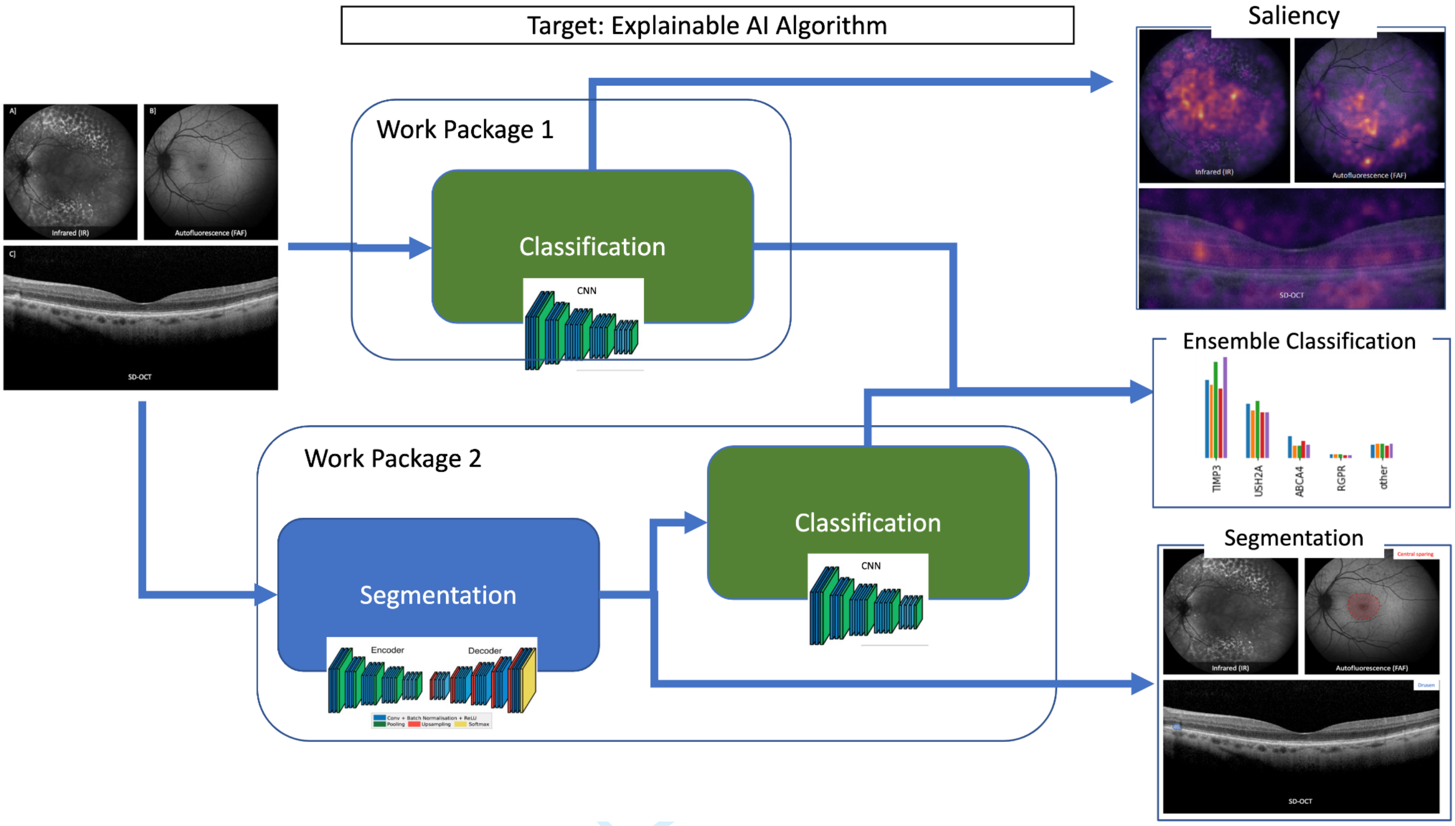

Supplementary Figure 3: By combining the classification network from WP1 with the segmentation network from WP2, Eye2Gene can provide highlight features used in the classification.

TRIPOD Checklist: Prediction Model Development

Section/Topic	Item	Checklist Item	Page
**Title and abstract**			
Title	1	Identify the study as developing and/or validating a multivariable prediction model, the target population, and the outcome to be predicted.	1
Abstract	2	Provide a summary of objectives, study design, setting, participants, sample size, predictors, outcome, statistical analysis, results, and conclusions.	3
**Introduction**			
Background and objectives	3a	Explain the medical context (including whether diagnostic or prognostic) and rationale for developing or validating the multivariable prediction model, including references to existing models.	4
	3b	Specify the objectives, including whether the study describes the development or validation of the model or both.	4
**Methods**			
Source of data	4a	Describe the study design or source of data (e.g., randomized trial, cohort, or registry data), separately for the development and validation data sets, if applicable.	7
	4b	Specify the key study dates, including start of accrual; end of accrual; and, if applicable, end of follow-up.	7
Participants	5a	Specify key elements of the study setting (e.g., primary care, secondary care, general population) including number and location of centres.	7
	5b	Describe eligibility criteria for participants.	8
	5c	Give details of treatments received, if relevant.	
Outcome	6a	Clearly define the outcome that is predicted by the prediction model, including how and when assessed.	10
	6b	Report any actions to blind assessment of the outcome to be predicted.	n/a
Predictors	7a	Clearly define all predictors used in developing or validating the multivariable prediction model, including how and when they were measured.	10
	7b	Report any actions to blind assessment of predictors for the outcome and other predictors.	n/a
Sample size	8	Explain how the study size was arrived at.	7
Missing data	9	Describe how missing data were handled (e.g., complete-case analysis, single imputation, multiple imputation) with details of any imputation method.	
Statistical analysis methods	10a	Describe how predictors were handled in the analyses.	10
	10b	Specify type of model, all model-building procedures (including any predictor selection), and method for internal validation.	9
	10d	Specify all measures used to assess model performance and, if relevant, to compare multiple models.	10
Risk groups	11	Provide details on how risk groups were created, if done.	n/a
**Results**			
Participants	13a	Describe the flow of participants through the study, including the number of participants with and without the outcome and, if applicable, a summary of the follow-up time. A diagram may be helpful.	7
	13b	Describe the characteristics of the participants (basic demographics, clinical features, available predictors), including the number of participants with missing data for predictors and outcome.	7
Model development	14a	Specify the number of participants and outcome events in each analysis.	7
	14b	If done, report the unadjusted association between each candidate predictor and outcome.	n/a
Model specification	15a	Present the full prediction model to allow predictions for individuals (i.e., all regression coefficients, and model intercept or baseline survival at a given time point).	n/a
	15b	Explain how to the use the prediction model.	n/a
Model performance	16	Report performance measures (with CIs) for the prediction model.	n/a
**Discussion**			
Limitations	18	Discuss any limitations of the study (such as nonrepresentative sample, few events per predictor, missing data).	n/a
Interpretation	19b	Give an overall interpretation of the results, considering objectives, limitations, and results from similar studies, and other relevant evidence.	n/a
Implications	20	Discuss the potential clinical use of the model and implications for future research.	n/a
**Other information**			
Supplementary information	21	Provide information about the availability of supplementary resources, such as study protocol, Web calculator, and data sets.	12
Funding	22	Give the source of funding and the role of the funders for the present study.	12

We recommend using the TRIPOD Checklist in conjunction with the TRIPOD Explanation and Elaboration document.

For peer review only - http://bmjopen.bmj.com/site/about/guidelines.xhtml

Can artificial intelligence accelerate the diagnosis of inherited retinal diseases? Protocol for a data-only retrospective cohort study (Eye2Gene)

Journal:	*BMJ Open*
Manuscript ID	bmjopen-2022-071043.R1
Article Type:	Protocol
Date Submitted by the Author:	05-Mar-2023
Complete List of Authors:	Nguyen, Quang; UCL Institute of Health Informatics,<br>Woof, William; Moorfields Eye Hospital NHS Foundation Trust; UCL Institute of Ophthalmology, Inherited Eye Diseases<br>Kabiri, Nathaniel; UCL Institute of Health Informatics<br>Sen, Sagnik; Moorfields Eye Hospital NHS Foundation Trust<br>Varela, Malena; Moorfields Eye Hospital NHS Foundation Trust; UCL Institute of Ophthalmology, Inherited Eye Diseases<br>Cabral De Guimaraes, Thales; UCL Institute of Ophthalmology, Inherited Eye Diseases; Moorfields Eye Hospital NHS Foundation Trust<br>Shah, Mital; Oxford Eye Hospital<br>Sumodhee, Dayyanah; Moorfields Eye Hospital NHS Foundation Trust<br>Moghul, Ismail; UCL Cancer Institute; Moorfields Eye Hospital NHS Foundation Trust<br>Al-Khuzaei, Saoud; Oxford Eye Hospital<br>Liu, Yichen; UCL Institute of Ophthalmology, Inherited Eye Diseases; Moorfields Eye Hospital NHS Foundation Trust<br>Hollyhead, Catherine; Eye2Gene Patient Advisory Group<br>Tailor, Bhavna; Eye2Gene Patient Advisory Group<br>Lobo, Loy; Eye2Gene Patient Advisory Group<br>Veal, Carl; Eye2Gene Patient Advisory Group<br>Archer, Stephen; Eye2Gene Patient Advisory Group<br>Furman, Jennifer; UCL<br>Arno, Gavin; UCL Institute of Ophthalmology, Inherited Eye Diseases<br>Gomes, Manuel; University College London, Department for Applied Health Research<br>Fujinami, Kaoru; Kankakuki Center<br>Madhusudhan, Savita; Royal Liverpool and Broadgreen University Hospitals NHS Trust<br>Mahroo, Omar; UCL Institute of Ophthalmology, Inherited Eye Diseases; Moorfields Eye Hospital NHS Foundation Trust<br>Webster, Andrew; University College London, Institute of Ophthalmology; Moorfields Eye Hospital NHS Foundation Trust<br>Balaskas, Konstantinos; Moorfields Eye Hospital NHS Foundation Trust, ; University of Manchester , School of Biological Sciences<br>Downes, Susan; Oxford Eye Hospital; Oxford Eye Hospital<br>Michaelides, Michel; Moorfields Eye Hospital NHS Foundation Trust; UCL Institute of Ophthalmology, Inherited Eye Diseases<br>Pontikos, Nikolas; University College London, Institute of Ophthalmology; Moorfields Eye Hospital NHS Foundation Trust

**Primary Subject Heading**:	Ophthalmology
Secondary Subject Heading:	Diagnostics, Health informatics, Genetics and genomics, Patient-centred medicine
Keywords:	STATISTICS & RESEARCH METHODS, OPHTHALMOLOGY, GENETICS

SCHOLARONE™
Manuscripts

Can artificial intelligence accelerate the diagnosis of inherited retinal diseases? Protocol for a data-only retrospective cohort study (Eye2Gene)

Quang Nguyen[1,2,9], William Woof[1,2], Nathaniel Kabiri[1,2], Sagnik Sen[2], Malena Daich Varela[1,2], Thales Antonio Cabral de Guimarães[1,2], Mital Shah[3,4], Dayyanah Sumodhee[2], Ismail Moghul[2], Saoud Al-Khuzaei[3,4], Yichen Liu[1,2], Catherine Hollyhead[8], Bhavna Tailor[8], Loy Lobo[8], Carl Veal[8], Stephen Archer[8], Jennifer Furman[10], Gavin Arno[1,2], Manuel Gomes[16], Kaoru Fujinami[5,6], Savita Madhusadhan[7], Omar A Mahroo[1,2], Andrew R Webster[1,2], Konstantinos Balaskas[1,2], Susan Downes[3,4], Michel Michaelides[1,2], Nikolas Pontikos[1,2]*

[1] University College London Institute of Ophthalmology, University College London, 11-43 Bath Street, London EC1V 9EL, UK
[2] Moorfields Eye Hospital, 162 City Road, London EC1V 2PD, UK
[3] Oxford Eye Hospital, John Radcliffe Hospital, Oxford OX3 9DU, UK
[4] Nuffield Department of Clinical Neurosciences, John Radcliffe Hospital, Oxford OX3 9DU, UK
[5] Laboratory of Visual Physiology, Division of Vision Research, National Institute of Sensory Organs, National Hospital Organization Tokyo Medical Center, Tokyo, Japan.
[6] Department of Ophthalmology, Keio University School of Medicine, Tokyo, Japan.
[7] St Paul's Eye Unit, The Royal Liverpool and Broadgreen University Hospitals, Liverpool L7 8XP, UK
[8] Eye2Gene Patient Advisory Group, London, UK
[9] University College London Institute of Health Informatics, 222 Euston Rd, London NW1 2DA, UK
[10] University College London Translational Research Office, 149 Tottenham Court Road, London W1T 7NF, UK
[11] University College London Institute of Epidemiology & Health, 1-19 Torrington Place, London WC1E 7HB, UK

*Correspondence to:
Nikolas Pontikos
n.pontikos@ucl.ac.uk

ORCID:

**Name**	**ORCID**
Mr Quang Nguyen	0000-0003-2665-3125
Dr William Woof, PhD	0000-0001-7555-329X
Mr Nathaniel Kabiri	0000-0003-3684-0020
Dr Sagnik Sen, MD	0000-0001-5835-5371
Dr Malena Daich Varela, MD	0000-0003-4960-4510
Dr Thales Antonio Cabral de Guimarães, MD	0000-0002-7936-6851
Dr Mital Shah, MD, PhD	0000-0001-7496-6121
Dr Dayyanah Sumodhee, DPsychol	0000-0001-7274-6088
Dr Ismail Moghul, PhD	0000-0003-3653-2327
Saoud Al-Khuzaei, MD	0000-0002-2779-3202
Mr Yichen Liu	0000-0002-9563-9151
Dr Jennifer Furman, PhD	0000-0002-1755-3774
Dr Gavin Arno, PhD	0000-0002-6165-7888
Dr Manuel Gomes, PhD	0000-0002-1428-1232
Prof Kaoru Fujinami, MD, PhD	0000-0003-4248-0033
Dr Savita Madhusudhan, MD	0000-0002-8203-0929
Prof Omar A Mahroo, MD, PhD	0000-0003-1254-0832
Prof Andrew R Webster, MD	0000-0001-6915-9560
Dr Konstantinos Balaskas, MD	0000-0003-2034-8920
Prof Susan Downes, MD	0000-0001-7373-2665
Prof Michel Michaelides, MD, PhD	0000-0002-1552-7046
Dr Nikolas Pontikos, PhD	0000-0003-1782-4711

IRAS: 242050
Data Protection Refence: Z6364106/2021/11/67

Word count: 3974

KEYWORDS: Artificial intelligence; Machine Learning; Retina; Eye Diseases, Hereditary; Blindness; Mass Screening; Vision Disorders

ABSTRACT

Introduction: Inherited retinal diseases (IRD) are a leading cause of visual impairment and blindness in the working age population. Mutations in over 300 genes have been seen to be associated with IRDs and identifying the affected gene in patients by molecular genetic testing is the first step towards effective care and patient management. However, genetics diagnosis is currently slow, expensive and not widely accessible. The aim of the current project is to address the evidence gap in IRD diagnosis with an AI algorithm, Eye2Gene, to accelerate and democratise the IRD diagnosis service.

Methods and analysis: The data-only retrospective cohort study involves a target sample size of 10,000 participants, which has been derived based on the number of participants with IRD at the three leading UK eye hospitals: Moorfields Eye Hospital (MEH), Oxford University Hospital, and Liverpool University Hospital, as well as a Japanese hospital, the Tokyo Medical Centre (TMC). Eye2Gene aims to predict causative genes from retinal images of patients with a diagnosis of IRD. For this purpose, 36 most common causative IRD genes have been selected to develop a training dataset for the software to have enough examples for training and validation of each gene detection. The Eye2Gene algorithm is composed of multiple deep convolutional neural networks, which will be trained on MEH IRD datasets, externally validated on OUH, LUH and TMC.

Ethics and dissemination: This research was approved by the IRB and the UK Health Research Authority (Research Ethics Committee reference 22/WA/0049) “Eye2Gene: accelerating the diagnosis of inherited retinal diseases” Integrated Research Application System (IRAS) project ID: 242050. All research adhered to the tenets of the Declaration of Helsinki. Findings will be reported in an open-access journal.

Strengths and limitations of this study

- One of the largest databases in the world of patients with inherited retinal disease who have undergone genetic screening and modern retinal imaging, analysed using novel artificial intelligence approaches.
- Robust evaluation and external validation at three different sites of an artificial intelligence algorithm on the task of automatically identifying up to 36 distinct genes from retinal in patient suspected to have an inherited retinal disease.
- Artificial intelligence performance is very dependent on the gene distribution of the training dataset, which is very imbalanced in the case for inherited retinal diseases, hence the need for external validation.

INTRODUCTION

The retina is the light-sensitive tissue at the back of our eyes which transforms light into electrical signals to the brain and is responsible for vision. The inherited retinal diseases (IRDs) are a group of diseases resulting from variation in proteins involved in retinal function. They represent the most common cause of blindness in young people in the UK and a leading cause of severe visual impairment and/or blindness in the working age population [1]. IRDs affect more than 2 million people globally and over 1 in 3000 people in the UK [2,3].

The age of disease onset varies with different IRDs, and patients usually have a progressive deterioration of their peripheral or central vision over several decades [4]. Hence, it is important to identify an IRD at an early stage, so that patients can undergo proper characterisation of the disease accurately. Treatments are emerging for some IRDs, but most are gene-specific, requiring identification of the precise causative genetic mutation [5,6].

Mutations in over 300 genes are associated with IRDs [7,8]. Identifying the causative gene is the first step towards diagnosis, prognosis and treatment. Currently, IRDs are usually detected first by community opticians and referred to ophthalmology for retinal imaging and diagnosis with a subsequent referral to specialist eye hospitals, such as Moorfields Eye Hospital, for further imaging and a genetic test.

However, due to limitations in the availability of IRD clinical expertise, detection and diagnostic rates remain poor, with most individuals having to wait for an average of 5.6 years in the UK for a diagnosis [9]. In addition, the diagnosis can cost the establishment £10,000 to obtain a final diagnosis for the patients and their families, starting from primary referral, to tertiary care, testing, investigation and genetic counselling [1,10]. Hence insufficient data on understanding of the disease prevalence and detection has contributed to insufficient funding available for testing of IRDs and associated counselling for patients and families. This delays development of possible treatment pathways and assistance with sight loss. As a result, a significant proportion of patients remain undiagnosed (**Figure 1**).

The proposal herein is to prepare images of historical IRD participant retinal scans (datasets) from eye hospitals located in the UK and in Japan:

- Moorfields Eye Hospital (MEH)
- Oxford University Hospital (OUH)
- Liverpool University Hospital (LUH)
- Tokyo Medical Centre (TMC)

Retinal scan datasets will be used to benchmark, train and test Eye2Gene, a deep-learning algorithm designed to detect and diagnose IRDs from a participant's retinal scan (**Figure 2**).

Aims and objectives

The aim of Eye2Gene is to provide detection and assist in diagnosis of IRDs through non-specialist centres within months instead of years. Eye2Gene does not aim to replace molecular diagnosis obtained through genetic testing, but it serves to narrow down the possibilities of genetic diagnosis based on imaging features, so that an early decision regarding patient care can be taken, and further testing offered after careful discussion with all stakeholders. It will also act as a tool for dissemination of expert IRD knowledge locally across the NHS.

By increasing the diagnostic rate for IRDs at a decreased cost, and by offering equitable access to a genetic diagnosis, the anticipated impacts for participants are:

- Improved health outcomes

- Earlier clinical diagnosis
- Personalised treatment plans (emerging treatments or clinical trials)
- Better understanding of the condition, its prognosis and its heritability for family planning

For the NHS:

- Improved operational efficiency both for the prescription and interpretation of genetic tests
- Increased genetic diagnostic rate at eye hospital
- Reduced economic burden by reducing the universality of genetic testing

The broad aim is to address the evidence gap in IRD diagnosis with an AI algorithm, Eye2Gene, to accelerate and increase availability of a specialist IRD diagnostic service at point of care.

Our primary objectives are training and further validation of Eye2Gene on independent datasets from three external sites: OUH, LUH and TMC, which include:

- To refine and improve our model, particularly with respect to rarer genes
- To provide explainability by identifying segmented IRD-specific features in classified images
- To investigate and develop saliency maps for our networks
- To validate Eye2Gene on external datasets to ensure it performs consistently well in different contexts (i.e that the model is generalisable)

Our secondary objectives are:

- To provide explainability by accurately identifying specific abnormalities (IRD-specific features) in retinal scans
- To lay the groundwork for development of Eye2Gene into a medical device

METHODS AND ANALYSIS

Work plan and timelines for delivery

Eye2Gene project will be divided into eight Work Packages (WP) (illustrated in **Figure 3**).

WP1: Development of Classification Algorithm

This will involve developing a Convolutional Neural Network model that can generalise to the N most common IRD genes at Moorfields and provide a top-5 accuracy of at least 88%. In particular, we will focus on achieve high per-gene accuracy for the rarer genes (which the current iteration of the model currently underpredicts). Additionally, part of this milestone will be to establish the value of N (the number of genes covered by the model) which we will pick based on all the data available across the four sites. We will assume N to be at least 10 for now, as this covers 70% of IRD cases and will be represented in the datasets of the four centres

WP2: Development of Segmentation Algorithm

This will involve the manually curated and segmented dataset provided by the Moorfields Reading Centre IRD Segmentation Team. The team will consist of graders and software developers under the lead of Dr Balaskas at the Moorfields Reading Centre. These segmented IRD datasets will be useful for the training of multiple AI algorithms including Eye2Gene. These will include a total of 14 retino-choroidal features detectable by Spectral Domain Optical Coherence Tomography (SD-OCT) or Fundus Auto-Fluorescence (FAF) or both, as well as their location, shape, and distribution. A segmentation algorithm based on U-Net [11] will be developed using this dataset.

WP3: Development of Explainable AI Algorithm

By combining the output of the classification algorithm (WP1) with the segmentation/classification algorithms (WP2), we will build an explainable AI algorithm that combines accuracy (WP1) and explainability (WP2). The final output of these models will be combined in a multinomial logistic regression with additional optional inputs such as age, sex, ethnicity, and mode of inheritance, to enhance predictive power. We will also be continuing to investigate and improve saliency maps for our models, and other explainability measures such as model confidence scores.

WP4: Phenotype-driven Genetic Variant Prioritisation

Deriving gene score based from the Eye2Gene classification gene probability from WP3. Also segmented IRD-features may be translated to Human Phenotype Ontology (HPO) terms in order to support HPO-base phenotype prioritisation using approaches such as Exomiser[12]. We will assess the utility of Eye2Gene for phenotype-driven variant prioritisation to help solve cases with multiple candidate variants. This will fulfil the ACMG annotation guidelines PP4 criteria, namely that the patient's phenotype or family history is highly specific for a disease with a single genetic aetiology [13].

WP5: Health Economic Evaluation

Health economic evaluation comparing the current treatment process to that of Eye2Gene will be conducted. The evaluation will consider two treatment pathways (standard care and the use of Eye2Gene), and will model resource use and cost, including the cost of validation, the cost of genetic tests, the time to find the genetics diagnosis (staff time), and the estimated cost of misdiagnosis, as well as the outcomes of standard and early diagnosis.

WP6: Eye2Gene Medical Software

Once we have completed the prototype as part of WP3, a software consultancy company (Phenopolis Ltd) will, under the oversight of regulatory consultants and the UCL Translational Research Office, develop Eye2Gene as medical device software following a QMS approach. In the first instance, the software will be developed to be hosted on a server that will likely be cloud-based.

WP7: Patient and Public Involvement

Patient Advisory Group (PAG) will feed into the decision making and the dissemination of results. The PAG will meet three times a year (January, May, September), each meeting will be 90 minutes and feed directly into the input of Eye2Gene. During this process, any risks raised by participants will be added to the risk register for the QMS.

WP8: Human Factors

User experience, usability and accessibility research will underpin the development of Eye2Gene. Following completion of WP3 we will have a working version of Eye2Gene to explore human factors around user expectations and experience.

Study design and population

This is an investigation aiming to develop an AI software as a medical device. It is a data-only retrospective cohort study that will utilise images (retinal scans), associated scan-specific (such as laterality, scan date and modality) and participant-specific (such as molecular diagnosis, mode of inheritance, age, and ethnicity) labels.

The study population includes data from participants that have received a IRD diagnosis, which has been molecularly confirmed via means of genetic test and have had retinal scans acquired using the Spectralis from Heidelberg Engineering (Dossenheim, Germany) with one of the following imaging modalities: IR, SD-OCT and FAF.

The study population at MEH has been derived by querying the OpenEyes Electronic Health Record (EHR) for IRD participants with a known genetic diagnosis and joining it up to the imaging databases of retinal scans (Heidelberg Medical Image Database) on hospital numbers. This enabled inclusion of all participants at MEH with an IRD who have both a genetic diagnosis and retinal scans available.

The study populations at OUH, LUH and TMC have been estimated based on information provided by the respective Principal Investigators, Prof Downes, Dr Madhusudhan and Prof Fujinami. This information has also been obtained by querying their local EHR databases and joining the dataset from the imaging database by hospital number.

Derivation of sample size

The target sample size of 10,000 participants has been derived based on the number of participants with IRD at the three leading UK eye hospitals participating in this study (MEH, OUH and LUH), as well as a Japanese hospital, the Tokyo Medical Centre (TMC). Given the rare nature of IRDs and that the study works on retrospectively collected anonymised data, we are targeting the largest datasets available in the UK.

The 36 most common genes have been selected as these should have enough training examples to ensure at least 10 example images for each fold, when split into 5 folds (after removing an initial held-out participant set). This is to ensure a meaningful amount of test data for each class per-fold when performing a 5-fold cross validation study. This also ensures at least 40 training images per class for each split, which is about the minimum number of training examples with which a CNN can still achieve good results [14].

Data acquisition

Participants will be identified by the care team of the respective site PIs by searching their medical records for patients who have received a molecularly confirmed genetic diagnosis for IRD. Data from MEH will be obtained by searching the EHR (OpenEyes) for participants with genetic reports entered in the EHR. The hospital numbers for these participants will be extracted and cross-referenced with the hospital numbers extracted from the imaging database, as shown in **Figure 4**. A similar approach will be undertaken at LUH, OUH and TMC to link the imaging data to the genetic reports and other associated metadata (age; mode of inheritance; and ethnicity) using the respective medical records in those sites and collating information from various spreadsheet, as needed.

Participants' data extracted from medical records and imaging databases at each site will be used to produce a list of images labelled with genes and metadata, where available. Data will be pseudonymised by the respective clinical teams, assigning a unique study ID to each patient, and keeping the link between each study ID and original hospital number at each of the respective sites. The study team working on developing the AI algorithm at UCL will not have access to the original hospital IDs. Following export, the images will be quality controlled as described in the **Inclusion criteria** section below and uploaded, for each patient, to the Moorfields Reading Centre data-transfer portal (grading.readingcentre.org).

Note that data collection for this study at each site is often an involved process since the data requires preparation, which needs to be overseen and carefully quality controlled by the site PI. Firstly, patient genetic information is not always in the EHR in a research-ready format but instead located in study spreadsheets. Therefore, various spreadsheets containing participant-level information including demographics and clinical information such as genetic diagnosis and phenotype, may need to be collated. Once the participant information has been prepared, their corresponding scans need to be extracted from the Heyex medical imaging database. Since most sites lack a Vendor Neutral Archive (unfortunately these are still rare in ophthalmology), this process requires cross-referencing of scans to participant, extracting them from the Heyex database as E2E files one patient at a time, and uploading

them to the Moorfields Reading Centre data-sharing platform (grading.readingcentre.org). These scans are then converted to an open format so they can be processed by the AI or annotated as part of WP2.

Consent/consent exemptions

The project is limited to the use of previously collected, non-identifiable information. As only anonymised clinical data will be made available to the research team and no study procedures will be carried out as part of this study, informed consent will not be separately sought from participants. However, consent will be obtained from the participants of the human factors research conducted as part of WP8 to gather user feedback on Eye2Gene.

Inclusion criteria

There will be no age restrictions for participants contributing data to train Eye2Gene, however it is anticipated that most will be over the age of 18. The inclusion criteria require participants to have both a confirmed IRD genetic diagnosis available that conforms to criteria (A) below and retinal imaging scan data available that conforms to criteria (B) below

(A) Criteria for IRD genetic diagnosis:

- An IRD genetic diagnosis consists of the identification of the IRD gene thought to be associated with the IRD condition of the participant.
- An IRD genetic diagnosis will often include the specific genetic variations which are thought to cause the disease.
- The IRD genetic diagnosis may have been conducted via a clinical NHS genetic testing service or through a research study.
- Both sources will be included in this study.

(B) Criteria for retinal imaging scan data:

Retina imaging scans will have been acquired with a medical imaging device (such as the Spectralis, Heidelberg) fixated on the macula and may belong to one of the following three categories:

- Fundus Auto-Fluorescence (FAF)
- Infrared (IR)
- Spectral Domain Optical Coherence Tomography (SD-OCT)

Image quality will be an important factor to consider. In order to assess image quality objectively, image quality scores such as the Blind Referenceless Image Spatial Quality Evaluator (BRISQUE) [15] image quality score will be applied. The criteria currently applied for image quality at MEH have been summarised in **Table 1.** These scan quality thresholds will be reviewed and potentially adjusted depending on the data quality available across sites.

Quality of the FAF scans	Quality of the IR scans	Quality of the OCT scans
BRISQUE score < 120	BRISQUE score < 80	BRISQUE score < 150
Median intensity > 0.05	Median intensity > 0.1	Max intensity < 1<br>OR<br>mean intensity < 0.2
“Noise level metric” (i.e. sum of square differences compared to blurred image via 5x5 box filter) < 2200		

Table 1. Scan quality criteria for images obtained at MEH to maintain minimum standards of inclusion into the study

Exclusion criteria

Participants that do not have a confirmed IRD genetic diagnosis or no retinal imaging data available. No other exclusion criteria apply.

Time period of data collection and follow-up

The data collection will happen in the first two years of the study (Jan 2022 – Jan 2024) to obtain retrospective observational data from all four sites. There will be no follow-up as all data is collected retrospectively for participants that have already received a genetic diagnosis. Following lead-in times, including ethics approvals, contractual procedures and data sharing agreements, data collection from UK sites (MEH, OUH and LUH) started in June 2022 and is likely to finish towards December 2023. Due to the additional challenges surrounding international data sharing and transfer arrangements, data collection at the Japanese site (TMC) was delayed to December 2022 and consequently, is likely to complete in January 2024. For the reasons explained above in 'Data Acquisition', the data collection, although retrospective, is a lengthy process which should finish by the end of 2023. In addition, as part of WP2, there is also an additional manual process undertaken of manually grading scans which will likely continue in the background for the entire duration of the project.

Description of collected data

Along with the gene diagnosis and the retinal scans, the following information will be collected where available:

- Site: MEH, OUH, LUH or TMC
- Scan metadata:
    - Laterality
    - Scan modality
    - Date scan was acquired
- Participant demographic data:
    - Age when scan was acquired
    - Biological sex
    - Ethnicity
- Clinical information pertinent to disease:
    - Mode of inheritance
    - Age of onset

All data will be consistently coded across sites and pseudonymised. A unique study ID will be assigned to each participant and the link between the study IDs and original hospital number identifiers will be kept at each of the respective sites and not shared with the research team.

Deep learning protocols

A Convolutional Neural Network (CNN) [16] will be used to classify the images. It will be trained on retinal images from patients with IRD labelled with the causative gene. The aim will be to input a previously unseen retinal image and output a prediction of the causative gene (WP1) (see **Supplementary Figure 1**).

Next, a subset of scans will be manually annotated, as part of WP2. This data will be used to train a U-Net [11], a commonly used neural network architecture for image segmentation tasks (**Supplementary**

Figure 2). These will include a total of 14 retino-choroidal features detectable by SD-OCT or FAF or both, as well as their location, shape, and distribution.

Specifically, on SD-OCT we will segment 8 features:

- Drusen
- Subretinal fluid
- Intraretinal fluid (cysts)
- Subretinal hyper-reflective material
- Ellipsoid zone loss
- Retinal pigment epithelium loss
- Choroidal hyper-transmission
- Foveal hypoplasia

On FAF/IR we will segment 6 features:

- Hypo/hyper autofluorescence patterns
- Drusen
- Flecks
- Peripapillary sparing
- Vessel attenuation
- Foveal hypo-autofluorescence loss

On the other hand, development of Deep Neural Network will also involve the manually curated and segmented dataset provided by the Moorfields Reading Centre IRD Segmentation Team. The team will consist of 4 graders and 2 software developers under the lead of an IRD expert at Moorfields and the director of the Moorfields Reading Centre.

Statistical methods and performance evaluation

We aim to develop a model that can generalise to the N most common IRD genes at Moorfields and provide a top-5 accuracy of at least 95%. In particular, we will focus on per-gene accuracy for the rarer genes (which the current iteration of the model currently underpredicts). Gene-specific or phenotype-specific segmentation features will be delineated in the Moorfields dataset and will be internally validated by the clinical team using the Dice similarity coefficient score [17]:

$$DSC = \frac{2\ |A \cap B|}{|A| + |B|}$$

where A and B are the regions defined by the two annotated features, to assess overlap with manual segmentation. Images with Dice score over 0.8 will be selected for training and validation.

The final output of classification and segmentation models will be combined in a multinomial logistic regression with additional optional inputs such as age, sex, ethnicity, and mode of inheritance, to enhance predictive power (**Supplementary Figure 3**). We will also be continuing to investigate saliency maps for our models, and other explainability measures such as model confidence scores.

The algorithm will be externally validated on the multisite data. This might require further calibration of parameter weights for age, sex, ethnicity, and mode of inheritance, per site. We will use top-1 and top-5 classification accuracy and mean per-gene defined as area under the receiver-operator curve (ROC AUC) score as the metrics for evaluation. We will also review the interpretation of the output (segmentation and saliency maps) qualitatively as part of Humans Factors (WP8). The data is stored at

https://grading.readingcentre.org and can be made available for viewing at the request of the editors/reviewers. The code can be found at https://github.com/Eye2Gene.

Measures to avoid bias

Site, age, gender, ethnicity, and mode of inheritance are all potential sources of bias. These will be fitted as extra covariables into the classification algorithm using a multinomial regression or another equivalent statistical method to avoid confounding. Since only retrospective data will be used in a first instance there will be no ascertainment bias as the data will represent routine IRD department activity at the respective hospitals.

A known source of bias in the data which cannot be corrected is the inherent imbalance in the data: more common diagnosis vs rarer diagnosis. The implications of this is that Eye2Gene will tend to overpredict the common classes and underpredict the rare classes.

Patient and public involvement

Participants will be engaged for service mapping; knowledge gathering; acceptability testing; and questionnaire design. A number of activities planned for the PAG and charity partners include:

- Interviews and focus groups
- Survey and report designs
- Data monitoring advice
- Dissemination of research

We will attend and present at participant days organised by charity partners, including Stargardt's Connected and RetinaUK. During these conferences, Eye2Gene will be presented to participants who will be invited to share their views on the project. In the third year, we will liaise with the Moorfields Patient Public Involvement (PPI) team to organise an Eye2Gene participant day. Participants will be invited to discuss their diagnostic experience and view a presentation of Eye2Gene.

As the project advances, participants will be given the opportunity to participate in one-to-one interviews and focus groups, aiming at an in-depth investigation of participant experience and feedback on Eye2Gene (WP8). During these interviews, participant experience surrounding the genetic diagnosis process will be further explored:

- Aspects of health psychological support and health anxiety
- Participant expectations about AI
- Education and genetic counselling
- Areas for improvement

Exploration of participant preferences (WP5) in year two (2023) will be formally quantified using a Discrete Choice Experiment (DCE) to investigate participants' preference in their diagnostic journey will help inform future research and implementation.

The Chief Investigator, Dr Pontikos, and co-investigators, supported by a health psychologist collaborator, Dr Sumodhee, with experience in qualitative research and genetic counsellors will gather patient feedback at various events. These include focus groups held once a year (September), a participant day in the third year (June 2024), and any others organised by the Moorfields PPI teams.

During these events, participant experiences surrounding the genetic diagnosis process will be further explored. In particular, aspects of health psychological support, doctor-participant communication when

discussing diagnosis and health anxiety, and participant education and genetic counselling. We will seek to understand how the current genetic diagnosis service experience can be improved with Eye2Gene. Preliminary results of PPI engagement can be found in **Appendix A.**

ETHICS AND DISSEMINATION

This research was approved by the IRB and the UK Health Research Authority (Research Ethics Committee (REC) reference 22/WA/0049) "Eye2Gene: accelerating the diagnosis of inherited retinal diseases" Integrated Research Application System (IRAS) (project ID: 242050). All research adhered to the tenets of the Declaration of Helsinki. Findings will be reported in an open-access journal.

Our PPI group will be involved in dissemination of the results. Additionally, our IRD participants that are signed up to our mailing lists will be informed of our progress.

STATEMENTS

Acknowledgements

As NIHR funded this study, publications will be published according to their guidelines. Moorfields Eye Charity has seed-funded part of this work, hence will be acknowledged in publications. Furthermore, the Archer family has also made a donation to the Eye2Gene project previously, hence will also be acknowledged.

Competing interests

Neither the chief investigator (Dr Pontikos) nor any of the co-investigators have any direct personal involvement (financial, shareholder or personal) with the NIHR that may give rise to a conflict of interest. As has been disclosed to the study sponsor (UCL) and the funder (NIHR), Dr Pontikos and Dr Moghul are shareholders at Phenopolis Ltd, which is one of the two subcontractors mentioned above (WP6). Phenopolis Ltd will provide professional software development services for Eye2Gene.

Funding

The work was funded by grant number AI_AWARD02488 from the National Institute for Health Research (NIHR) Biomedical Research Centre (BRC) at Moorfields Eye Hospital NHS Foundation Trust, and UCL Institute of Ophthalmology.

Contributors

The chief investigator NP conceptualised the Eye2Gene study, designed and developed the protocol. NP, QN, YL and WW contributing to deep learning protocols. SS, TAC, MV, SAK, KF, OM, MM, AW, KB, SD, SM, contributed to reviewing the clinical background. GA contributed to writing the phenotype-driven interpretation work package. MG contributed to writing the health economics work package. IM and MS contributed to writing the software as a medical device work package. DS, NK, WW and the Eye2Gene Patient Advisory Group (CH, BT, LL, CV and SA) contributed to writing the Patient and Public Involvement work package. DS contributed to writing the human factors work package. JF and SA contributed to writing the project management work package. NP contributed to all work packages. All authors contributed to critically reviewing and formatting the protocol. All authors approved the final version of the manuscript for submission for publication.

REFERENCES

[1] Galvin O, Chi G, Brady L, Hippert C, Del Valle Rubido M, Daly A, et al. The Impact of Inherited Retinal Diseases in the Republic of Ireland (ROI) and the United Kingdom (UK) from a Cost-of-Illness Perspective. Clin Ophthalmol 2020;14:707–19. https://doi.org/10.2147/OPTH.S241928.
[2] Berger W, Kloeckener-Gruissem B, Neidhardt J. The molecular basis of human retinal and vitreoretinal diseases. Prog Retin Eye Res 2010;29:335–75. https://doi.org/10.1016/j.preteyeres.2010.03.004.
[3] Hanany M, Rivolta C, Sharon D. Worldwide carrier frequency and genetic prevalence of autosomal recessive inherited retinal diseases. Proc Natl Acad Sci U S A 2020;117:2710–6. https://doi.org/10.1073/pnas.1913179117.
[4] Chen T-C, Huang D-S, Lin C-W, Yang C-H, Yang C-M, Wang VY, et al. Genetic characteristics and epidemiology of inherited retinal degeneration in Taiwan. NPJ Genom Med 2021;6:16. https://doi.org/10.1038/s41525-021-00180-1.
[5] Georgiou M, Fujinami K, Michaelides M. Inherited retinal diseases: Therapeutics, clinical trials and end points-A review. Clin Experiment Ophthalmol 2021;49:270–88. https://doi.org/10.1111/ceo.13917.
[6] Varela MD, de Guimaraes TAC, Georgiou M, Michaelides M. Leber congenital amaurosis/early-onset severe retinal dystrophy: current management and clinical trials. Br J Ophthalmol 2022;106:445–51. https://doi.org/10.1136/bjophthalmol-2020-318483.
[7] Duncan JL, Pierce EA, Laster AM, Daiger SP, Birch DG, Ash JD, et al. Inherited Retinal Degenerations: Current Landscape and Knowledge Gaps. Transl Vis Sci Technol 2018;7:6. https://doi.org/10.1167/tvst.7.4.6.
[8] Lázaro-Guevara JM, Flores-Robles BJ, Garrido K, Pinillos-Aransay V, Elena-Ibáñez A, Merino-Meléndez L, et al. Gene's hubs in retinal diseases: A retinal disease network. Heliyon 2018;4:e00867. https://doi.org/10.1016/j.heliyon.2018.e00867.
[9] The Lancet Diabetes Endocrinology. Spotlight on rare diseases. Lancet Diabetes Endocrinol 2019;7:75. https://doi.org/10.1016/S2213-8587(19)30006-3.
[10] Mendelian. A preliminary assessment of the potential impact of rare diseases on the NHS. 2018.
[11] Ronneberger O, Fischer P, Brox T. U-net: Convolutional networks for biomedical image segmentation. Lecture Notes in Computer Science (including subseries Lecture Notes in Artificial Intelligence and Lecture Notes in Bioinformatics), vol. 9351, Springer Verlag; 2015, p. 234–41. https://doi.org/10.1007/978-3-319-24574-4_28.
[12] Jacobsen JOB, Kelly C, Cipriani V, Robinson PN, Smedley D. Evaluation of phenotype-driven gene prioritization methods for Mendelian diseases. Brief Bioinform 2022;23. https://doi.org/10.1093/bib/bbac188.
[13] Richards S, Aziz N, Bale S, Bick D, Das S, Gastier-Foster J, et al. Standards and guidelines for the interpretation of sequence variants: a joint consensus recommendation of the American College of Medical Genetics and Genomics and the Association for Molecular Pathology. Genet Med 2015;17:405–23.
[14] Cho J, Lee K, Shin E, Choy G, Do S. How much data is needed to train a medical image deep learning system to achieve necessary high accuracy? ArXiv [CsLG] 2015.
[15] Mittal A, Moorthy AK, Bovik AC. Blind/Referenceless Image Spatial Quality Evaluator. 2011 Conference Record of the Forty Fifth Asilomar Conference on Signals, Systems and Computers (ASILOMAR), 2011, p. 723–7. https://doi.org/10.1109/ACSSC.2011.6190099.
[16] Lecun Y, Bengio Y, Hinton G. Deep learning. Nature 2015;521:436–44. https://doi.org/10.1038/nature14539.
[17] Dice LR. Measures of the Amount of Ecologic Association Between Species. Ecology 1945;26:297–302. https://doi.org/10.2307/1932409.

FIGURE LEGENDS

Figure 1. A summary of the inherited retinal disease patient population in the United Kingdom. On average, it takes over 5 years and approximately £10,000 for patients and families of patients to receive

a final genetic diagnosis. Of the 30,000 individuals with inherited retinal disease, over a third have not yet received a genetic diagnosis.

Figure 2. Eye2Gene supports the three main retinal imaging modalities: **(A)** Infrared (IR) **(B)** Autofluorescence (FAF) **(C)** Spectral domain optical coherence tomography (SD-OCT).

Figure 3. An overview of main work packages (WP) for Eye2Gene.

Figure 4. A data flow diagram summarising the extraction of data from Moorfields Eye Hospital and the external sites (OUH; LUH; and TMC); secure transfer to the Moorfields Eye Hospital and UCL secure databases; and processing, to train and validate the Eye2Gene system.

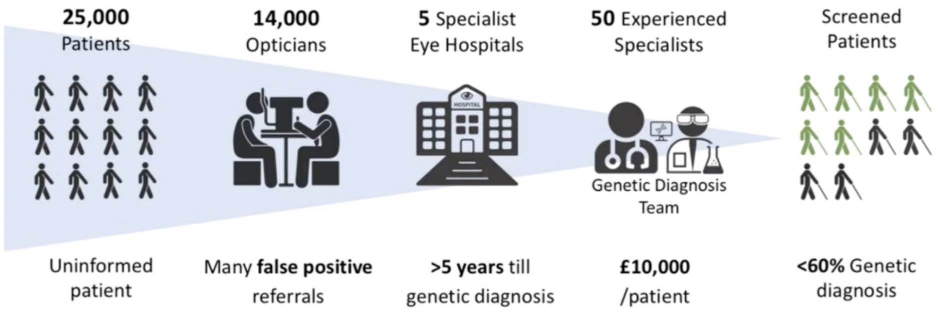

A summary of the inherited retinal disease patient population in the United Kingdom. On average, it takes over 5 years and approximately £10,000 for patients and families of patients to receive a final genetic diagnosis. Of the 30,000 individuals with inherited retinal disease, over a third have not yet received a genetic diagnosis.

383x136mm (144 x 144 DPI)

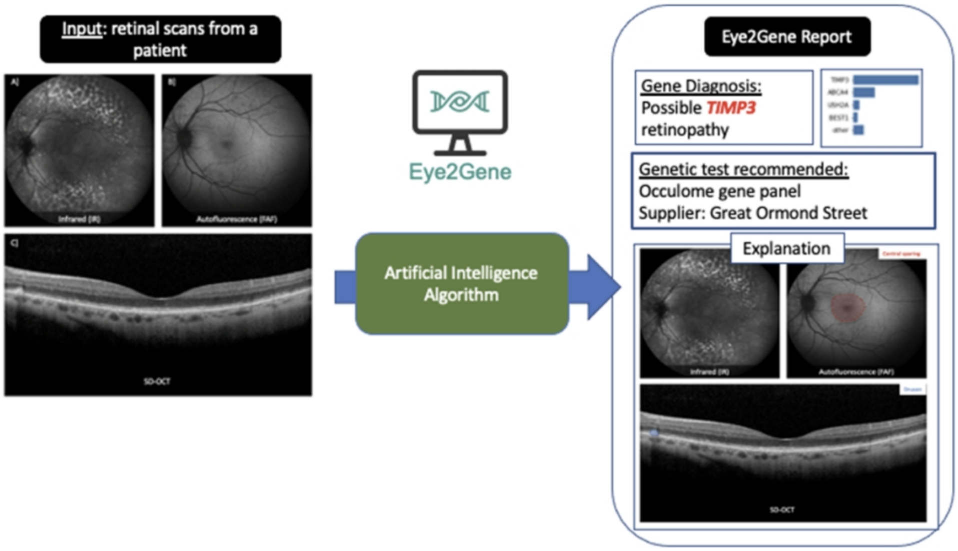

Eye2Gene is an AI algorithm that rapidly recognises the gene associated with an inherited retinal disease, accelerating the genetic diagnosis. Eye2Gene supports the three main retinal imaging modalities: (A) Infrared (IR) (B) Autofluorescence (FAF) (C) Spectral domain optical coherence tomography (SD-OCT).

524x301mm (144 x 144 DPI)

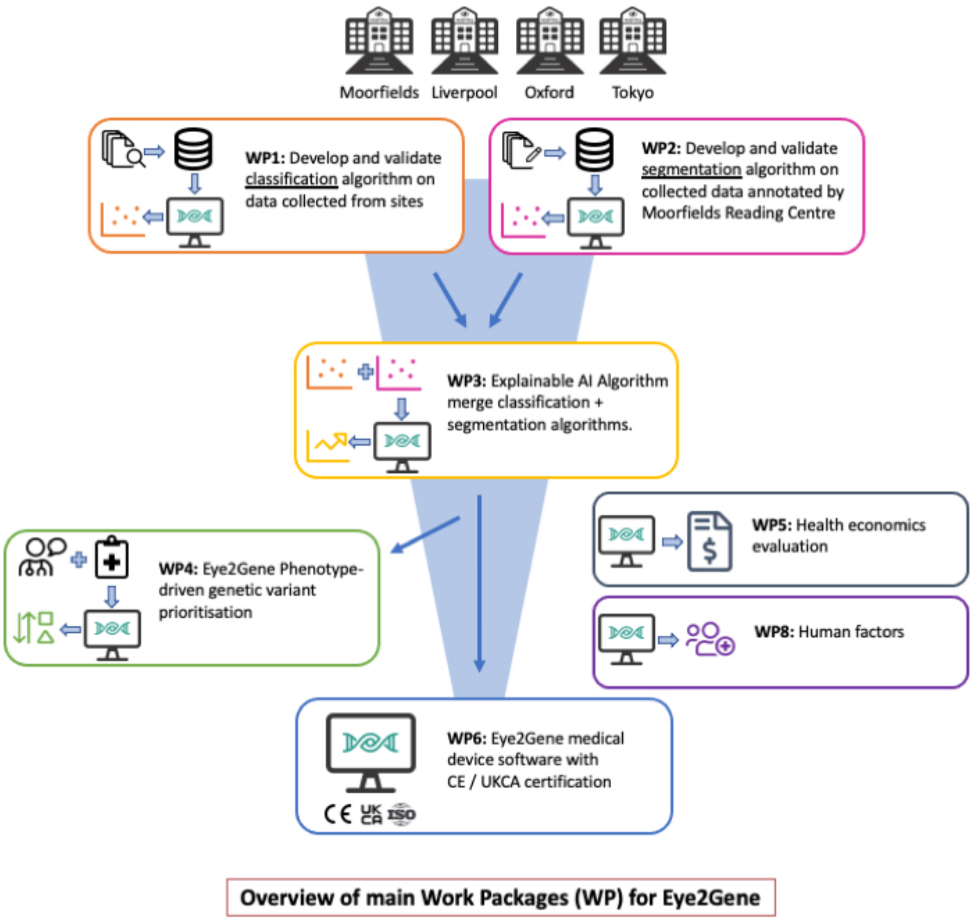

An overview of main work packages (WP) for Eye2Gene.

358x358mm (51 x 51 DPI)

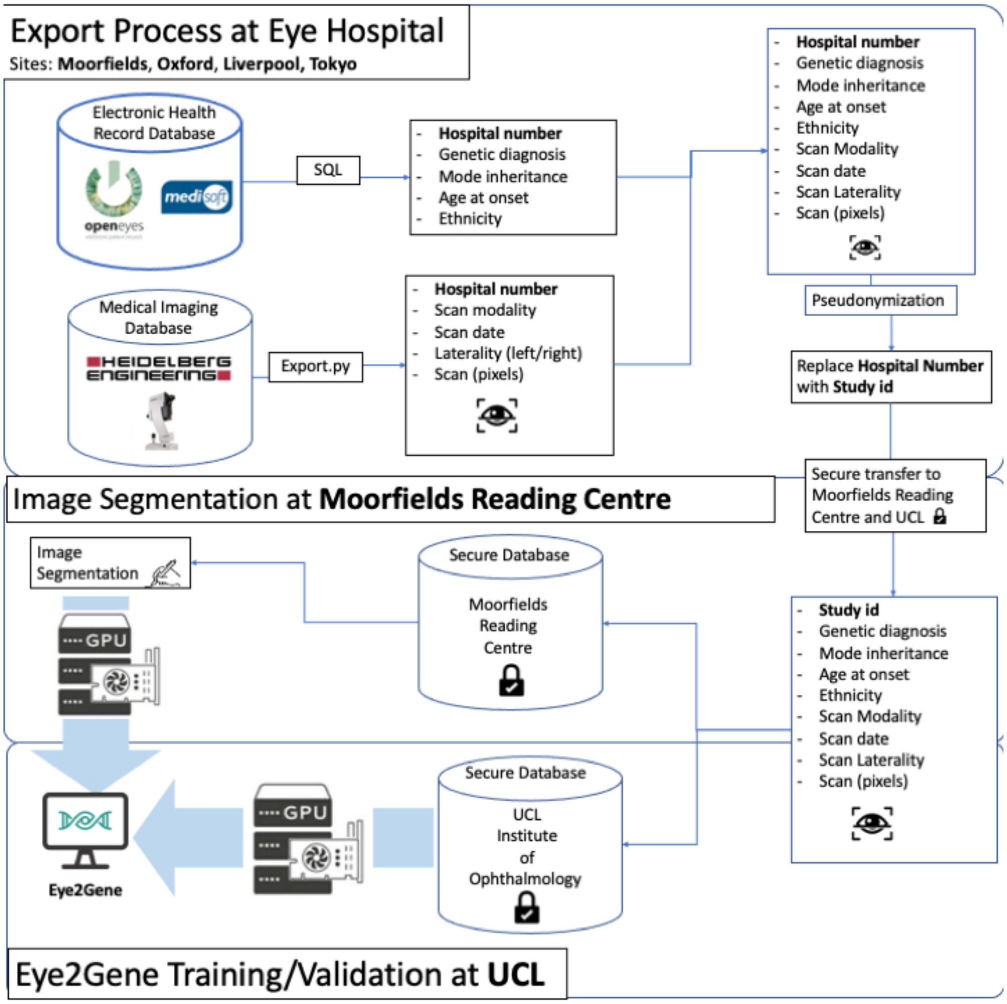

A data flow diagram summarising the extraction of data from Moorfields Eye Hospital and the external sites (OUH; LUH; and TMC); secure transfer to the Moorfields Eye Hospital and UCL secure databases; and processing, to train and validate the Eye2Gene system.

358x358mm (51 x 51 DPI)

APPENDIX

A. PPI - Contributions to research design from patients

Patients have helped design and review the study protocol and have accepted the research. Acceptability and feasibility have been assessed as part of Phase 1 conducted through patient days with Moorfields (HDRUK-funded) and focus groups (NIHR PPIE enabling fund). In 2019, two focus groups and a patient day were conducted to explore IRD patient needs, as part of the HDRUK-funded MyEyeSite project.

When patients were interviewed to assess acceptability and feasibility of Eye2Gene to assist IRD diagnosis, our mixed-methods research (Gilbert et al., 2022) found that 82% wanted to be engaged in managing their own health data. Reasons given included:

- "To obtain genetic testing information for an affected child, or for fertility/genetics counselling family planning".
- "To participate in an international clinical trial".
- "Out of curiosity or personal interest in my condition".
- "To support a claim for personal independence payment".
- "To share data with another hospital (e.g., for diagnosing deafness or for cancer treatment)".

Further to this, an NIHR RDS Enabling Involvement Fund (awarded on the 12th of August 2020) allowed for the recruitment of 6 patients to review the Eye2Gene project, in addition to a further 4 who volunteered and waived compensation.

Two teleconference events with focus groups were organised in 2020, to provide feedback on the Eye2Gene proposal and the research programme. The first was held on the 21st of August and attended by 5 participants. The second, held on the 3rd of September, was attended by a further 5 participants. Both meetings were summarised in note form and moderated by a health psychologist collaborator. The major outcomes of the focus groups were:

- Patients suggested changes to the text to improve readability to a lay audience
- Patients clarified their needs and expectations of the project
- Patients suggested extending Eye2Gene to advise on potential treatments

Five patients volunteered to collaborate on the project, and have committed to specific roles as part of the Patient Advisory Group (PAG)

For peer review only

SUPPLEMENTARY FIGURES

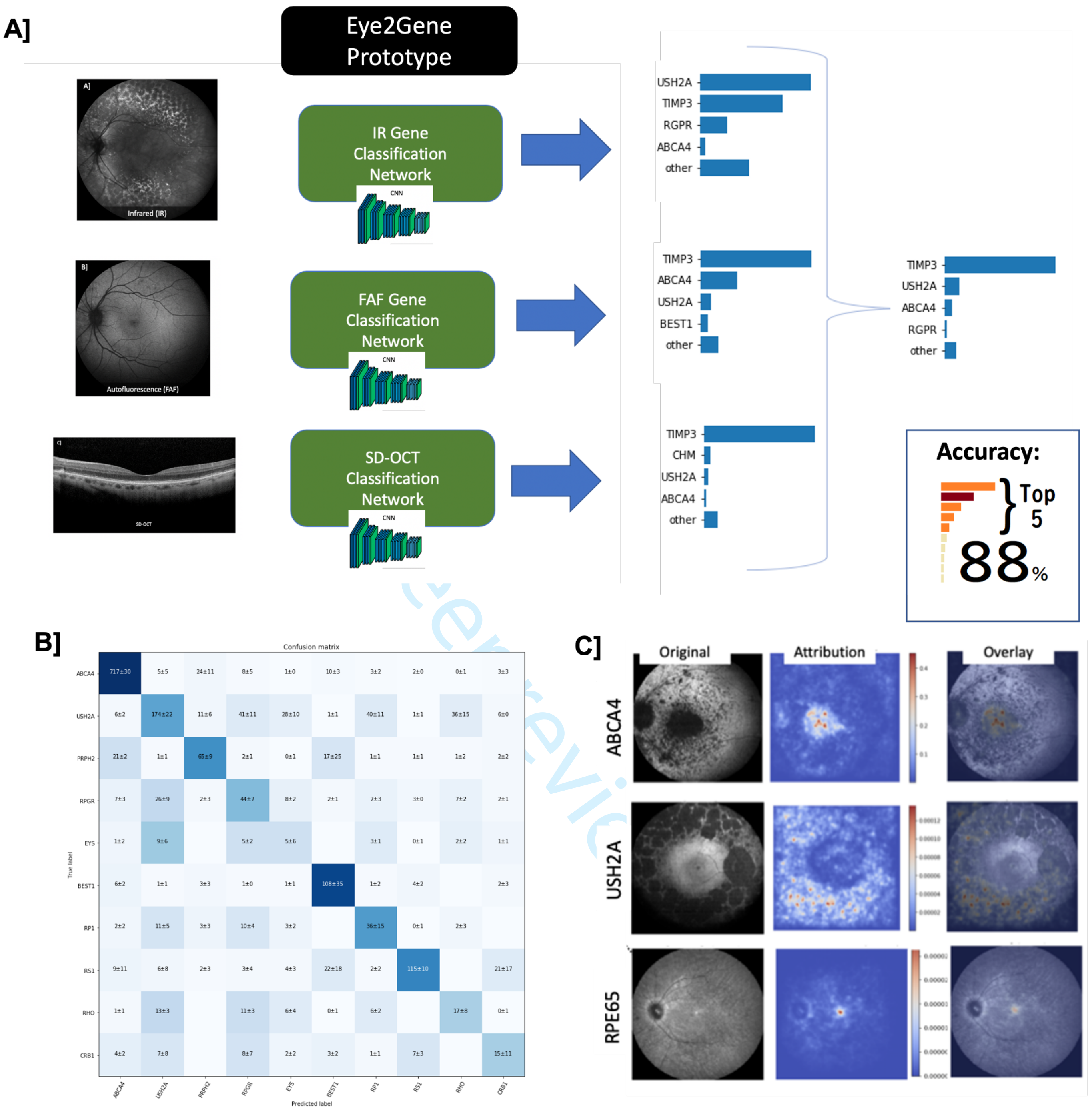

Supplementary Figure 1: A) The Eye2Gene prototype is able to provide an IRD-gene prediction given a retinal scan of one of the three imaging modalities (FAF; IR; and SD-OCT) (WP1). The top-5 accuracy of Eye2Gene is 88%. **B)** Confusion matrix indicating the misclassification errors for the top 10 genes. **C)** Attribution maps for FAFs indicate which pixels are deemed important by the network in reaching a classification. Cone-rod and macular dystrophies activate central pixels in the fovea such as ABCA4 and RPE65 whereas rod-cone dystrophies such as USH2A activate pixels in the periphery.

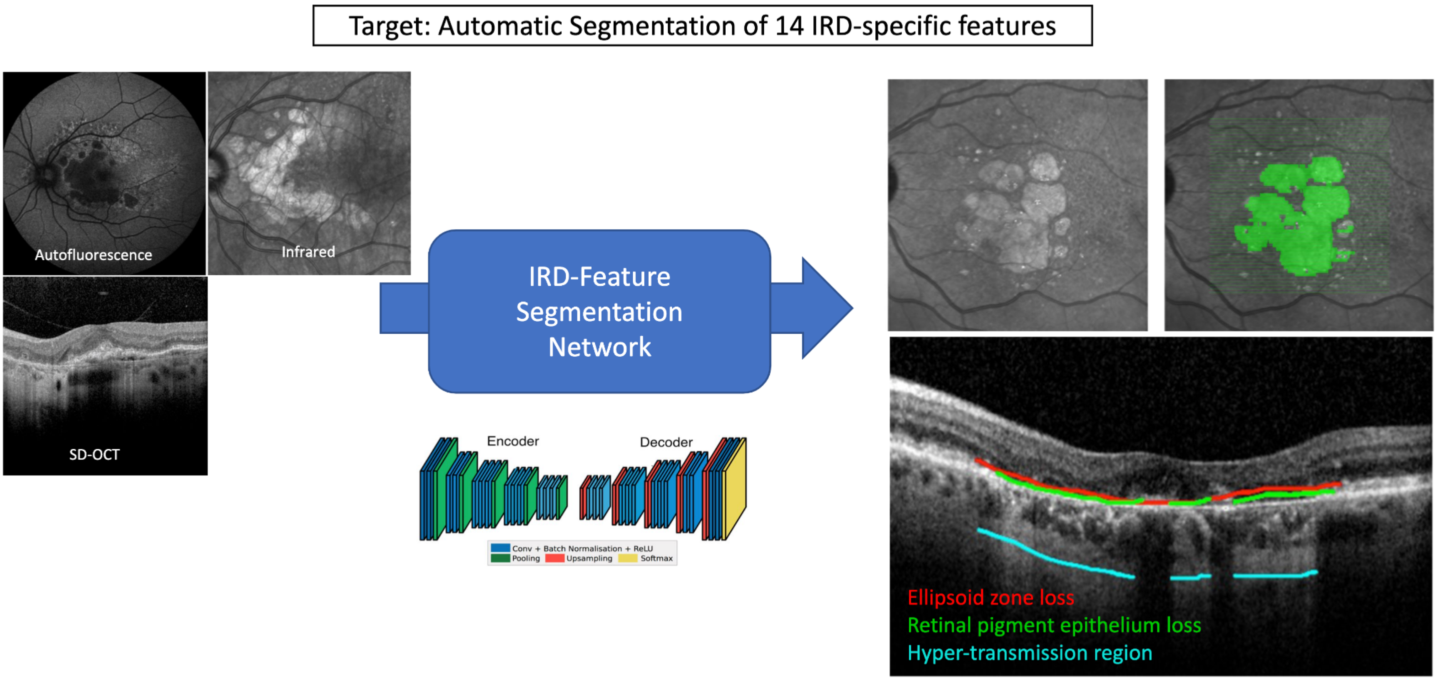

Supplementary Figure 2: The U-net architecture is characterized by an encoder-decoder structure. The encoder shares many similarities with classification networks, and aggregates information from a large spatial context into an abstract representation. From this abstract representation, the decoder subsequently reconstructs an image with the original resolution in which the output value for each pixel represents the segmentation label. This will be trained to segment the 14 features defined in WP2.

For peer review only

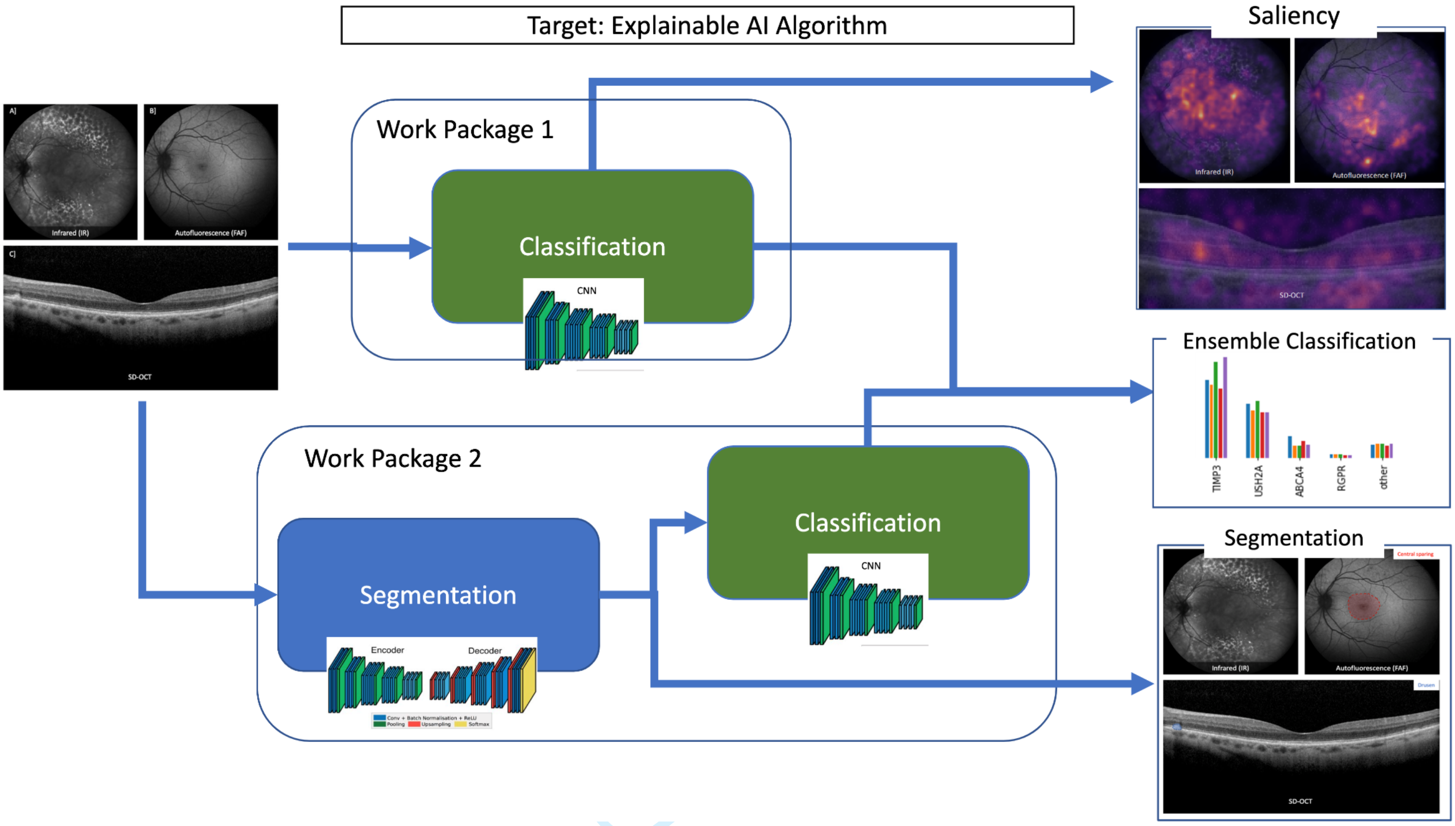

Supplementary Figure 3: By combining the classification network from WP1 with the segmentation network from WP2, Eye2Gene can provide highlight features used in the classification.

TRIPOD Checklist: Prediction Model Development

Section/Topic	Item	Checklist Item	Page
**Title and abstract**			
Title	1	Identify the study as developing and/or validating a multivariable prediction model, the target population, and the outcome to be predicted.	1
Abstract	2	Provide a summary of objectives, study design, setting, participants, sample size, predictors, outcome, statistical analysis, results, and conclusions.	3
**Introduction**			
Background and objectives	3a	Explain the medical context (including whether diagnostic or prognostic) and rationale for developing or validating the multivariable prediction model, including references to existing models.	4
	3b	Specify the objectives, including whether the study describes the development or validation of the model or both.	4
**Methods**			
Source of data	4a	Describe the study design or source of data (e.g., randomized trial, cohort, or registry data), separately for the development and validation data sets, if applicable.	7
	4b	Specify the key study dates, including start of accrual; end of accrual; and, if applicable, end of follow-up.	7
Participants	5a	Specify key elements of the study setting (e.g., primary care, secondary care, general population) including number and location of centres.	7
	5b	Describe eligibility criteria for participants.	8
	5c	Give details of treatments received, if relevant.	
Outcome	6a	Clearly define the outcome that is predicted by the prediction model, including how and when assessed.	10
	6b	Report any actions to blind assessment of the outcome to be predicted.	n/a
Predictors	7a	Clearly define all predictors used in developing or validating the multivariable prediction model, including how and when they were measured.	10
	7b	Report any actions to blind assessment of predictors for the outcome and other predictors.	n/a
Sample size	8	Explain how the study size was arrived at.	7
Missing data	9	Describe how missing data were handled (e.g., complete-case analysis, single imputation, multiple imputation) with details of any imputation method.	
Statistical analysis methods	10a	Describe how predictors were handled in the analyses.	10
	10b	Specify type of model, all model-building procedures (including any predictor selection), and method for internal validation.	9
	10d	Specify all measures used to assess model performance and, if relevant, to compare multiple models.	10
Risk groups	11	Provide details on how risk groups were created, if done.	n/a
**Results**			
Participants	13a	Describe the flow of participants through the study, including the number of participants with and without the outcome and, if applicable, a summary of the follow-up time. A diagram may be helpful.	7
	13b	Describe the characteristics of the participants (basic demographics, clinical features, available predictors), including the number of participants with missing data for predictors and outcome.	7
Model development	14a	Specify the number of participants and outcome events in each analysis.	7
	14b	If done, report the unadjusted association between each candidate predictor and outcome.	n/a
Model specification	15a	Present the full prediction model to allow predictions for individuals (i.e., all regression coefficients, and model intercept or baseline survival at a given time point).	n/a
	15b	Explain how to the use the prediction model.	n/a
Model performance	16	Report performance measures (with CIs) for the prediction model.	n/a
**Discussion**			
Limitations	18	Discuss any limitations of the study (such as nonrepresentative sample, few events per predictor, missing data).	n/a
Interpretation	19b	Give an overall interpretation of the results, considering objectives, limitations, and results from similar studies, and other relevant evidence.	n/a
Implications	20	Discuss the potential clinical use of the model and implications for future research.	n/a
**Other information**			
Supplementary information	21	Provide information about the availability of supplementary resources, such as study protocol, Web calculator, and data sets.	12
Funding	22	Give the source of funding and the role of the funders for the present study.	12

We recommend using the TRIPOD Checklist in conjunction with the TRIPOD Explanation and Elaboration document.
