# Supplementary material for: Can artificial intelligence accelerate the diagnosis of inherited retinal diseases? Protocol for a data-only retrospective cohort study (Eye2Gene)
Source: BMJ Open. 2023 Mar 20;13(3):e071043. doi: 10.1136/bmjopen-2022-071043 (PMC10030964; doi:10.1136/bmjopen-2022-071043)
Supplement: Reviewer comments [file bmjopen-2022-071043.reviewer_comments.pdf]

PEER REVIEW HISTORY

BMJ Open publishes all reviews undertaken for accepted manuscripts. Reviewers are asked to complete a checklist review form (**http://bmjopen.bmj.com/site/about/resources/checklist.pdf**) and are provided with free text boxes to elaborate on their assessment. These free text comments are reproduced below.

ARTICLE DETAILS

**TITLE (PROVISIONAL)**	Can artificial intelligence accelerate the diagnosis of inherited retinal diseases? Protocol for a data-only retrospective cohort study (Eye2Gene)
**AUTHORS**	Nguyen, Quang; Woof, William; Kabiri, Nathaniel; Sen, Sagnik; Varela, Malena; Cabral De Guimaraes, Thales; Shah, Mital; Sumodhee, Dayyanah; Moghul, Ismail; Al-Khuzaei, Saoud; Liu, Yichen; Hollyhead, Catherine; Tailor, Bhavna; Lobo, Loy; Veal, Carl; Archer, Stephen; Furman, Jennifer; Arno, Gavin; Gomes, Manuel; Fujinami, Kaoru; Madhusudhan, Savita; Mahroo, Omar; Webster, Andrew; Balaskas, Konstantinos; Downes, Susan; Michaelides, Michel; Pontikos, Nikolas

This article was not externally reviewed at BMJ Open. Protocols that have been independently assessed prior to submission to BMJ Open are usually fast-tracked to publication on the grounds that further substantial changes will not be possible. This independent assessment will usually be external, independent review for both a substantial grant award from a non-commercial or government funder and ethics approval.
